# Regulation of cyclic lipopeptide orfamide A biosynthesis in *Pseudomonas protegens* by the Gac–Rsm–LuxR Cascade

**DOI:** 10.1093/nar/gkag501

**Published:** 2026-05-20

**Authors:** Ruoyi Wang, Chenjie Yu, Yan Song, Lulu Zhang, PingPing Li, Linli Zhu, Yali Fu, Rongshi Chen, Guohua Ding, Rainer Borriss, Xuewen Gao, Junqing Qiao, Qin Gu, Huijun Wu

**Affiliations:** The Sanya Institute of Nanjing Agricultural University, Nanjing Agricultural University, 572024 Sanya, China; State Key Laboratory of Agricultural and Forestry Biosecurity, College of Plant Protection, Nanjing Agricultural University, 211800 Nanjing, China; The Sanya Institute of Nanjing Agricultural University, Nanjing Agricultural University, 572024 Sanya, China; State Key Laboratory of Agricultural and Forestry Biosecurity, College of Plant Protection, Nanjing Agricultural University, 211800 Nanjing, China; College of Horticulture Technology, Suzhou Polytechnic Institute of Agriculture, 215008 Suzhou, China; The Sanya Institute of Nanjing Agricultural University, Nanjing Agricultural University, 572024 Sanya, China; State Key Laboratory of Agricultural and Forestry Biosecurity, College of Plant Protection, Nanjing Agricultural University, 211800 Nanjing, China; The Sanya Institute of Nanjing Agricultural University, Nanjing Agricultural University, 572024 Sanya, China; State Key Laboratory of Agricultural and Forestry Biosecurity, College of Plant Protection, Nanjing Agricultural University, 211800 Nanjing, China; The Sanya Institute of Nanjing Agricultural University, Nanjing Agricultural University, 572024 Sanya, China; State Key Laboratory of Agricultural and Forestry Biosecurity, College of Plant Protection, Nanjing Agricultural University, 211800 Nanjing, China; The Sanya Institute of Nanjing Agricultural University, Nanjing Agricultural University, 572024 Sanya, China; State Key Laboratory of Agricultural and Forestry Biosecurity, College of Plant Protection, Nanjing Agricultural University, 211800 Nanjing, China; The Sanya Institute of Nanjing Agricultural University, Nanjing Agricultural University, 572024 Sanya, China; State Key Laboratory of Agricultural and Forestry Biosecurity, College of Plant Protection, Nanjing Agricultural University, 211800 Nanjing, China; The Sanya Institute of Nanjing Agricultural University, Nanjing Agricultural University, 572024 Sanya, China; State Key Laboratory of Agricultural and Forestry Biosecurity, College of Plant Protection, Nanjing Agricultural University, 211800 Nanjing, China; Modern Agricultural Analysis and Testing Center, Nanjing Agricultural University, 211800 Nanjing, China; Institute of Biology, Humboldt University, 10115 Berlin, Germany; The Sanya Institute of Nanjing Agricultural University, Nanjing Agricultural University, 572024 Sanya, China; State Key Laboratory of Agricultural and Forestry Biosecurity, College of Plant Protection, Nanjing Agricultural University, 211800 Nanjing, China; Institute of Plant Protection, Jiangsu Academy of Agricultural Sciences, 210014 Nanjing, China; The Sanya Institute of Nanjing Agricultural University, Nanjing Agricultural University, 572024 Sanya, China; State Key Laboratory of Agricultural and Forestry Biosecurity, College of Plant Protection, Nanjing Agricultural University, 211800 Nanjing, China; The Sanya Institute of Nanjing Agricultural University, Nanjing Agricultural University, 572024 Sanya, China; State Key Laboratory of Agricultural and Forestry Biosecurity, College of Plant Protection, Nanjing Agricultural University, 211800 Nanjing, China

## Abstract

Orfamide A, a lipopeptide produced by *Pseudomonas protegens* Pf-5, is a key determinant of its biocontrol properties. In this study, we investigated the regulatory interactions among the GacS/A two-component system, small RNAs (sRNAs), repressor proteins, and two LuxR-type transcription factors in orfamide A biosynthesis. We found that GacS/A indirectly regulates orfamide A production by enhancing transcription of three sRNAs (RsmX, RsmY, and RsmZ). RsmY and RsmZ synergistically relieve repression by RsmA and RsmE, while RsmX plays a lesser role, likely counteracting only one repressor. LuxR-type transcription factors, LuxR1 and LuxR2, which positively regulate orfamide A synthesis, are directly repressed by RsmA and RsmE via binding to their 5′ untranslated regions, linking them to the Gac–Rsm signaling cascade. We further demonstrated that LuxR2 activates *luxR1* expression, which in turn facilitates orfamide A production by binding to the promoter of the orfamide A biosynthetic gene cluster. Importantly, we showed that this entire regulatory cascade operates in the rhizosphere and directly influence biocontrol efficacy. These findings provide a comprehensive understanding of the Gac–Rsm–LuxR pathway in orfamide A biosynthesis and offer valuable insights for the development of biocontrol agents based on *Pseudomonas* strains.

## Graphical Abstract

**Graphical Abstract:**
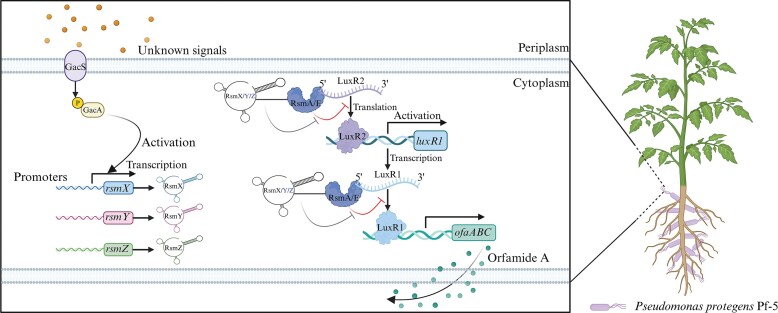

## Introduction

Plant growth-promoting rhizobacteria (PGPR) contribute to plant health by producing secondary metabolites that enhance growth and suppress plant pathogens [1, 2]. The *Pseudomonas fluorescens* group are well-characterized PGPR with broad applications in agriculture, such as *P. protegens* Pf-5 and CHA0 [3, 4]. Early work by Thomashow *et al*. revealed that the phenazine-1-carboxylic acid (PCA) produced by *P. fluorescens* 2–79 acted as an antibiotic against *Gaeumannomyces graminis*, the causative agent of take-all disease in wheat [5]. Subsequent studies by Nielsen *et al*. identified viscosinamide produced by *P. fluorescens* DR54 exerts suppressive effects on the hyphal growth of *Rhizoctonia solani* and *Pythium ultimum* [6]. Marcella D found that 2,4-diacetylphloroglucinol (DAPG) and pyoluteorin (Plt) of *P. protegens* Pf-5 exhibit antifungal properties [7]. Rezzonico *et al*. demonstrated the role of hydrogen cyanide from *P. protegens* CHA0 in suppressing *Fusarium oxysporum* f. sp. *radicis-lycopersici* (FORL) [8]. Together, these studies highlight the critical role of *Pseudomonas* secondary metabolites in disease suppression and plant–microbe interactions.

Bacteria utilize numerous two-component systems (TCSs), such as BarA/UvrY in *Escherichia coli*, BarA/SirA in *Salmonella*, VarS/VarA in *Vibrio*, and GacS/GacA in *Pseudomonas*, to sense and respond to environmental cues, orchestrate diverse physiological processes, including chemotaxis, virulence, nutrient acquisition, plant growth promotion, and the biosynthesis of secondary metabolites [9]. The Gac/Rsm cascade, consisting of GacS/A TCS, small RNAs (sRNAs), and repressor proteins [10], is widely conserved in *Pseudomonas* spp. [11–13]. sRNAs are key post-transcriptional regulators involved in bacterial metabolism, virulence, biofilm formation, and quorum sensing [14, 15]. In *Pseudomonas* spp., a subset of sRNAs acts by sequestering RNA-binding proteins of the RsmA/CsrA family [16–18], which inhibit translation by binding to the ribosome-binding site (RBS) of target mRNAs [19–21].

Orfamides are cyclic lipopeptides (CLPs) produced by *Pseudomonas* spp. via nonribosomal peptide synthases (NRPSs), exhibiting broad-spectrum antifungal and insecticidal activity [22, 23]. Among various orfamide homologues, orfamide A is the predominant variant synthesized by *P. protegens* and has been implicated in swarming motility, root colonization, and biocontrol efficacy [24, 25]. While orfamide biosynthesis is known to be regulated by the GacS/A, the downstream regulatory mechanisms remain poorly understood. Notably, NRPS gene clusters in *Pseudomonas* often co-localize with *luxR* or *luxR*-like transcription factor genes [26]. The *P. protegens* Pf-5 genome encodes two such LuxR-like regulators, LuxR1 and LuxR2; however, their roles and interactions in orfamide A regulation have not been elucidated.

Here, we show that GacS/A activates transcription of three sRNAs—RsmX, RsmY, and RsmZ—in Pf-5. RsmY and RsmZ relieve translational repression by both RsmA and RsmE, whereas RsmX antagonizes only one repressor. We further demonstrate that LuxR2 regulates luxR1 expression and that LuxR1 directly activates orfamide A biosynthesis. These findings uncover a hierarchical regulatory circuit linking the Gac/Rsm cascade to LuxR-type transcriptional control of orfamide production in *P. protegens* Pf-5.

## Materials and methods

### Strains, culture conditions, nucleotides, and chemical compounds

Strains and plasmids are described in Supplementary Table S1. *P. protegens* strain Pf-5 and its derivatives were grown at 28°C with shaking at 180 rpm in King’s B (KB) broth. All plasmids involved in experiments were hosted in *E. coli*, and cultivated in Luria-Bertani (LB) broth at 37°C with shaking at 180 rpm. Antibiotics were used at the indicated concentrations: 100 μg/ml ampicillin 50 μg/ml kanamycin, 25 μg/ml gentamicin, and 25 μg/ml tetracycline. The oligonucleotide primers, RNA probes, and DNA probes were described in Supplementary Table S2. Orfamide A was purchased from Sigma–Aldrich (St. Louis, MO, USA), which was >99% pure, as determined by high-performance liquid chromatography (HPLC) and liquid chromatography-tandem mass pectrometry (LC-MS), and was solubilized in methanol.

### Construction of Pf-5 mutants and derivatives

Gene knockout was constructed by using the homologous double‐crossover method with the suicide vector pK18mobsacB [27] and confirmed by PCR and sequence analysis. The downstream and upstream fragments were amplified from Pf-5 genomic DNA and were then used as templates to generate fused fragments, which were inserted into pK18mobsacB. The recombinant pK18mobsacB plasmids were then transformed into the wild‐type Pf-5 strain or into the single, double, or triple mutant, if necessary, and cultured in KB agar containing 50 μg/ml kanamycin and 100 μg/ml ampicillin. The single colonies were transferred to KB plates containing 10% sucrose to get rid of the pK18mobsacB suicide plasmid. The single colonies growing on sucrose plates were then cultured in plates containing either 50 μg/ml kanamycin and 100 μg/ml ampicillin together or 100 μg/ml ampicillin alone. Loss of the kanamycin resistance indicated successful double‐crossovers, which were further confirmed by PCR and sequencing. To generate the complementary strains, the corresponding gene fragments were amplified from the Pf-5 genome and cloned into relevant vectors, which were then transferred into the mutants. The primers and plasmids used in this study are listed in Supplementary Tables S1 and S2, respectively.

### Swarming motility assay

Swarming motility assay was conducted on KB medium with 0.7% BD Bacto^™^ agar. Briefly, strains were cultivated overnight and subsequently diluted (1:100) into fresh KB broth containing 100 μg/ml ampicillin. They were cultivated until the OD_600_ reached 1.0. Then, 5 μl of the bacterial suspension was spotted onto the center of semisolid agar medium. After incubation for a period of 6 to 8 h, observations and imaging were performed. For exogenous orfamide A assays, the compound was incorporated into the agar medium prior to pouring.

### Extraction and quantification of orfamide A

WT Pf-5 and its derivatives were grown overnight and then diluted (1:100) into 200 ml of fresh KB broth for 48 h at 28°C with shaking at 180 rpm. Sterilized resin XAD16N (Sigma) was added to the culture during fermentation to absorb compounds. Afterward, resin XAD16N was redissolved with methanol and incubated at 28°C for 4 h. The extracts were concentrated using a rotary evaporator, and the crude extract of metabolites was obtained by suspending in methanol and filtered (0.22 μm nylon). Then, the crude extracts were analyzed by HPLC chromatography for detecting the production of orfamide A synthesized by WT Pf-5 and its derivatives. Orfamide A was detected using the C18 chromatographic column (ZORBAX SB-C18, 4.6 × 250 mm) with a flow rate of 1 ml/min, an injection volume of 5 μl, and a UV detection wavelength of 220 nm. The mobile phase used in the study included A (HPLC-grade acetonitrile containing 0.1% trifluoroacetic acid) and B (Milli-Q water containing 0.1% trifluoroacetic acid). The HPLC procedure was a gradient elution from 5% A to 90% A within 20 min, and then holding 90% A for 20 min. Orfamide A eluted at 33.4 min.

### RNA-seq

*Pseudomonas* strains were grown overnight at 28°C and 180 rpm, and then transferred (1:100) into fresh KB broth for 12 h. Then, 2 ml cultures were collected and washed with PBS buffer (137 mM NaCl, 2.7 mM KCl, 10 mM Na_2_HPO_4_, 2 mM KH_2_PO_4_, pH 7.4) for three times. The total RNA was extracted and sequenced by Gene Denovo Biotechnology Co., Ltd. (Guangzhou, China). The low-quality reads were removed and the clean reads were mapped to the reference genome of *P. protegens* Pf-5. The differential expressed genes (DEGs) between two samples were analysed using the DESeq2 with a threshold of |log_2_(Fold Change)| >1 and FDR < 0.05. The Kyoto Encyclopedia of Genes and Genomes (KEGG) pathway analysis was applied to investigate the role of DEGs.

### Luciferase, β-galactosidase, and GFP activity assays

The promoters of *rsmX, rsmY, rsmZ, luxR1, luxR2*, and *ofaABC* were amplified and fused with the *lux* gene of pME6522 to generate the transcriptional reporters, and the promoters of *luxR1* and *luxR2* and *ofaABC* were fused with the *gfp* gene of pME6522 to generate the translational reporters. These reporters were then transformed into Pf-5 and its derivatives. These transcriptional reporters were streaked out on KB plates for 12 h, and subsequently sprayed with the luciferase assay agent, and then placed in dark for 5 min. The luciferase intensity was observed by VIBER Molecular Imaging System (Fusion FX7, France). For luciferase activity, these strains were cultured in KB medium, and the cultures were collected, and lysed by using the luciferase cell culture lysis reagents. A 50 μl volume of the luciferase assay substrates at room temperature were then added into 10 µl cell lysates to measure the relative light units by multifunctional microplate detector (Synergy H1, Biotek, USA). For GFP intensity, these transcriptional reporters were cultured overnight and 50 μl suspensions were measured by multifunctional microplate detector (Synergy H1, Biotek, USA). For measuring the β-galactosidase activities, the promoters of *luxR1, luxR2*, and *ofaABC* were amplified and fused with the *lacZ* gene of pME6015. These reporters were transformed into those strains, and the β-galactosidase activities were assessed by the Miller method [28] using ONPG as substrates.

### Construction of protein expression plasmids and protein purification

For the prokaryotic expression of recombinant proteins, coding sequences of *rsmA, rsmE, luxR1*, and *luxR2* genes were amplified from the Pf-5 genomic DNA. The *rsmA, rsmE* sequences were recombined into pET28a plasmids, and *luxR1* and *luxR2* were cloned into the pOPTHis plasmids. All constructs were confirmed by sequencing. These expression plasmids were transformed into *E. coli* BL21 (DE3) strains and cultivated in LB medium overnight. The bacterial suspensions were then transferred into fresh LB medium to culture until the OD_600_ reached 0.4–0.6. Protein expression was subsequently induced by the addition of 0.25 mM isopropyl-β-d-1-thiogalactopyranoside (IPTG), and the cultures were incubated at 16°C for at least 18 h. Cells were harvested by centrifugation and lysed by sonication in PBS buffer to generate the crude extract of recombinant proteins. The recombinant protein was purified by affinity chromatography using a His trap column (GE Healthcare, Chicago, USA) and desalted using a desalting column (GE Healthcare, Chicago, USA). The protein concentration was examined using the Bradford assay (Bio-Rad), and the proteins were stored at −80°C.

### *In vitro* transcription of 5′ untranslated regions

The *rsmX, rsmY* and *rsmZ* were PCR-amplified from the Pf-5 genome, with the upstream primers designed to incorporate a T7 promoter (TAATACGACTCACTATAGGG). Transcripts were synthesized *in vitro* with T7 High Yield RNA Transcription Kit (Vazyme) following the manufacturer’s instructions. The primers used in this study are listed in Supplementary Table S2. The *luxR1* and *luxR2* 5′UTR were synthesized by Sangon Biotech (Shanghai, China).

### Electrophoretic mobility shift assay (EMSA)

For RNA–EMSA, non-denaturant polyacrylamide gels (6%) were prepared using a mixture comprising 8.88 ml of nuclease-free water, 3 ml of 30% acrylamide, 5 ml of 10 × TBE (900 mM Tris, 900 mM Tris Boric acid, 20 mM Na_2_EDTA, pH 8.3), 112.5 µl of 10% APS, and 9.75 µl of TEMED. Prior to sample loading, the gels were pre-electrophoresed in 0.5 × TBE buffer at 100 V for 30–60 min. The biotin-labeled RNA was labeled by Pierce^™^ RNA 3′ End Biotinylation Kit (Thermo Scientific). Binding reactions (20 µl) contained nuclease-free water, 2 µl of 10 × EMSA binding buffer, 2 µl of 50% glycerol, 2 µl of 10 mg/ml tRNA, unlabeled target RNA, protein, and biotin-labeled target RNA. To minimize non-specific interactions, the mixtures were first incubated at room temperature for 10 min before the biotin-labeled RNA probes were added, and then followed by an additional incubation of 20–30 min. Subsequently, the reaction mixtures were fractionated on the non-denaturant polyacrylamide gel at 100 V, which was then transferred to the nylon membrane with positive charges at low temperature, 400 mA for 30–45 min. The nylon membrane was immediately exposed under a 254 nm UV-cross-linked bulb for 1 min at 120 mJ/cm^2^. The RNA–protein complexes were visualized by chemiluminescence as described in the LightShift^®^ Chemiluminescent RNA EMSA Kit (Thermo Scientific).

For DNA–EMSA, the non-denaturant polyacrylamide gels (6%) were composed of 13.2 ml of ddH_2_O, 4 ml of 30% acrylamide, 5 ml of 10 × TBE, 625 µl of 80% glycerol, 150 µl of 10% APS, and 10 µl of TEMED. The unlabeled and biotin-labeled DNA probes were amplified from the Pf-5 genome. The binding reactions (20 µl) contained protein, ddH_2_O, 2 µl of 10 × binding buffer, 1 µl of Poly (dl·dC), and biotin-labeled and unlabeled target probes. All subsequent steps followed the same procedure as described for RNA–EMSA.

### Microscale thermophoresis analysis (MST)

Direct interactions of RsmA and RsmE protein with *rsmX, rsmY, rsmZ*, and the 5′UTR RNA of *luxR1* and *luxR2* were measured by microscale thermophoresis using the Large Volume Protein Labeling Kit Red-tris-NTA 2^nd^ Generation (NanoTemper Technologies, Germany). The purified RsmA and RsmE protein were adjusted to an approximate concentration of 10 μM using label buffer (130 mM NaHCO_3_, 50 mM NaCl, pH 8.2–8.3). The resuspended RsmA and RsmE proteins were subsequently labeled by RED‐tris‐NTA 2^nd^‐generation dye at room temperature for 30 min.

For the interaction between LuxR1 and LuxR2 with the promoters of *luxR1* and *ofaABC*, the reporters represented the promoter of *luxR1* and *ofaABC* with FAM label. The ligand molecules were diluted using 0.1% PBS-T (137 mM NaCl, 2.7 mM KCl, 10 mM Na_2_HPO_4_, 2 mM KH_2_PO_4_, pH 7.4, 0.1% Tween-20) and incubated with an equal volume to the labeled proteins at room temperature for 5 min. The samples were then loaded into the Monolith NT.115 Capillaries, and the interactions were measured with 40% MST power in the Monolith NT.115 instrument (Nano Temper Technologies, Germany) by NanoTemper Monolith NT.115. MO. Affinity Analysis (x86) Software Version 2.3 was used to fit curves and calculate the value of the dissociation constants (*K*_d_).

### ChIP-qPCR analysis

ChIP experiments were performed as described previously with some modifications [29, 30]. Briefly, fresh bacterial pellets of Pf-5 (pME6015-GFP), Pf-5 (pME6015-*luxR1*-GFP), and Pf-5 (pME6015-*luxR2*-GFP) were cross-linked with 1% formaldehyde for 10 min, and the mixture was stopped with 125 mM glycine for 5 min. The samples were subsequently washed by Tris buffer (1 M Tris–HCl, pH 7.5, 5 M NaCl) for two times and re-suspended in lysis buffer (0.5 M HEPES-KOH, pH 7.5, 5 M NaCl, 0.5 M EDTA, pH 8.0, 1% Triton X-100, 10% SDS, and 5 mM sodium deoxycholate) with 1% lysozyme. DNA was sheared into 100–500 bp fragments with 30 s-on and 1 min-off by the Covaris E220 DNA Sonicator (Covaris, Woburn, MA) for a total on-time of 480 s. The lysates were centrifuged (12 000 × *g*, 10 min, 4°C) and digested with RNase for 30 min. The immunoprecipitation was incubated with the monoclonal anti-GFP (ab290, Abcam, Cambridge, UK) antibody and the protein A agarose beads (sc-2003, Santa Cruz, CA, USA) at 4°C for 8 h. The input samples were placed at −20°C. The antibody–bead complexes were successively washed with lysis buffer and TE buffer (1 M Tris–HCl, pH 8.0, 0.5 M EDTA, pH 8.0). After washing, 100 μl of elution buffer I (1 M Tris–HCl, pH 8.0, 0.5 M EDTA, pH 8.0, 10% SDS) was applied for eluting the DNA at 65°C for 20 min, and the beads were subsequently added with 150 μl of elution buffer II (TE buffer with 10% SDS). All samples were reverse cross-linked overnight and removed all proteins with proteinase K. DNA was extracted and used for ChIP-qPCR analysis. The PCR primers of ChIP-qPCR assays are listed in Supplementary Table S2. Relative enrichment values were calculated by dividing the amount of immunoprecipitated DNA by the amount of input DNA.

### Quantitative reverse transcription PCR

For real-time quantitative PCR (qRT-PCR), the strains were cultivated in KB medium, and total RNA was extracted using the bacterial RNA extraction kit (OMEGA Bio-tek, Inc. Norcross, GA, USA) and determined with the Nanodrop 8000 spectrophotometer. Afterward, the purified RNA was reverse transcribed using HiScript III RT SuperMix (Vazyme Biotech Co., Ltd., Nanjing, China). qRT-PCR was performed using the SYBR Green qPCR master mix (TakaraBio, Changping District, Beijing, China) in 96-well plates on the ABI QuantStudio^™^ 5 machine (ThermoFisher Scientific, Waltham, USA) with software version 2.3. The cycling conditions were 95°C/30 s and then 40 cycles of 95°C/5 s, 60°C/15 s. The comparative cycle threshold (CT) method was used for data analysis and relative fold difference was estimated as 2^−ΔΔCT^. The *gyrB* gene expression was employed as the internal control to normalize mRNA levels. Primer sequences used for qRT-PCR are listed in Supplementary Table S2.

### Plant disease control

Thirty-day-old tomato seedlings (variety cultivar “Mao fen”) roots were inoculated with 15 ml of *P. protegens* cultures (1 × 10^7^ CFU/ml), and 15 ml of *Ralstonia solanacearum* suspensions (1 × 10^7^ CFU/ml) were irrigated into the tomato seedlings at the next day. The control group was inoculated with *R. solanacearum* only. Each treatment contained 12 seedlings was applied in three separated experiments. All seedlings were maintained in the greenhouse (30°C, 75% relative humidity, 14 h of light/10 h of darkness), and inspected periodically until disease symptoms appeared. The disease severity was evaluated according to the following scale: Level 0 = plants without visible symptoms; Level 1 = striped necrosis on stems occasionally or less than half of the leaves wilted on unilateral stems; Level 2 = black streaks less than half the height of the stem or between half to two-thirds of the leaves wilted on unilateral stems; Level 3 = more than two-thirds of the leaves wilted on unilateral stems; Level 4 = the plant is dead [31, 32]. The disease severity index was calculated according to the following formula: Disease severity index (DSI, 100%)={∑ (X*Y)/(*Z* × 4) × 100%, Disease control effect (%)={(DSI_Control_ − DSI_Treatment_)/DSI_Control_}*100%. Where *X* = number of different degrees infected plants in the treatments; *Y* = relative degree value; and *Z* = number of total plants in the treatments.

### Matrix-assisted laser desorption/ionization time-of-flight mass spectrometry imaging (MALDI-MSI)

The preparation programs were based on previously reported methods [33–35]. For the MSI analysis detecting the orfamide A production by *P. protegens* on MM Medium (3.4 g/l KH_2_PO_4_, 5.08 g/l NH_4_Cl, 9.356 g/l monosodium L-glutamate, 29.715 g/l sodium succinic hexahydrate, 2.5% glycerol/20% glucose; adding 5 mM MgSO_4_ and 18 μM FeSO_4_ after sterilization), the indium tin oxide (ITO) glass microscope slide was placed between two layers of MM solid medium. The *Pseudomonas* strains were grown overnight and transferred in fresh KB medium until OD_600 _= 1.0. The cultures were subsequently inoculated onto the ITO slide and incubated overnight at 30°C. Carefully remove the ITO slide, dry it, and conduct the MSI analysis. For the MSI analysis of tomato roots, the sterilized tomato seeds were soaked in *Pseudomonas* cultures for 30 min and sown on water agar plates containing 1/2 sodium carboxymethyl cellulose (CMC). Plates were cultured in greenhouse at 30°C for 5–7 days until the seedlings emerged. The tomato roots were cut and frozen by liquid nitrogen to preserve metabolites and spatial structure, and subsequently embedded by 2% CMC. The frozen samples were sliced using a cryoslicer (CM1950, Leica, Germany) with a slice thickness of 10–20 μm, which were then adhered to ITO glass microscope slide. MALDI-MSI was carried out on Bruker UltrafleXtreme MALDI-TOF mass spectrometer (Bruker Daltonics, Bremen, Germany). The images were acquired using Bruker’s *FlexImaging* v. 6.0.

### Statistical analysis

All experiments were performed with at least three independent biological replicates (*n* = 3). Data are presented as mean ± standard deviation (SD). Statistical analysis was performed using SPSS software (SPSS Inc.). For multiple group comparisons, homogeneity of variances was confirmed by Levene’s test (*P* > 0.05). One-way analysis of variance (ANOVA) followed by Duncan’s multiple range test was used to determine statistically significant differences among groups. A value of *P* < 0.05 was considered statistically significant. For comparisons involving only two groups, Student’s *t*-test was used instead of ANOVA. A value of *P* < 0.05 was considered statistically significant.

## Results

### GacS/A regulates the production of orfamide A in Pf-5

**Figure 1.**
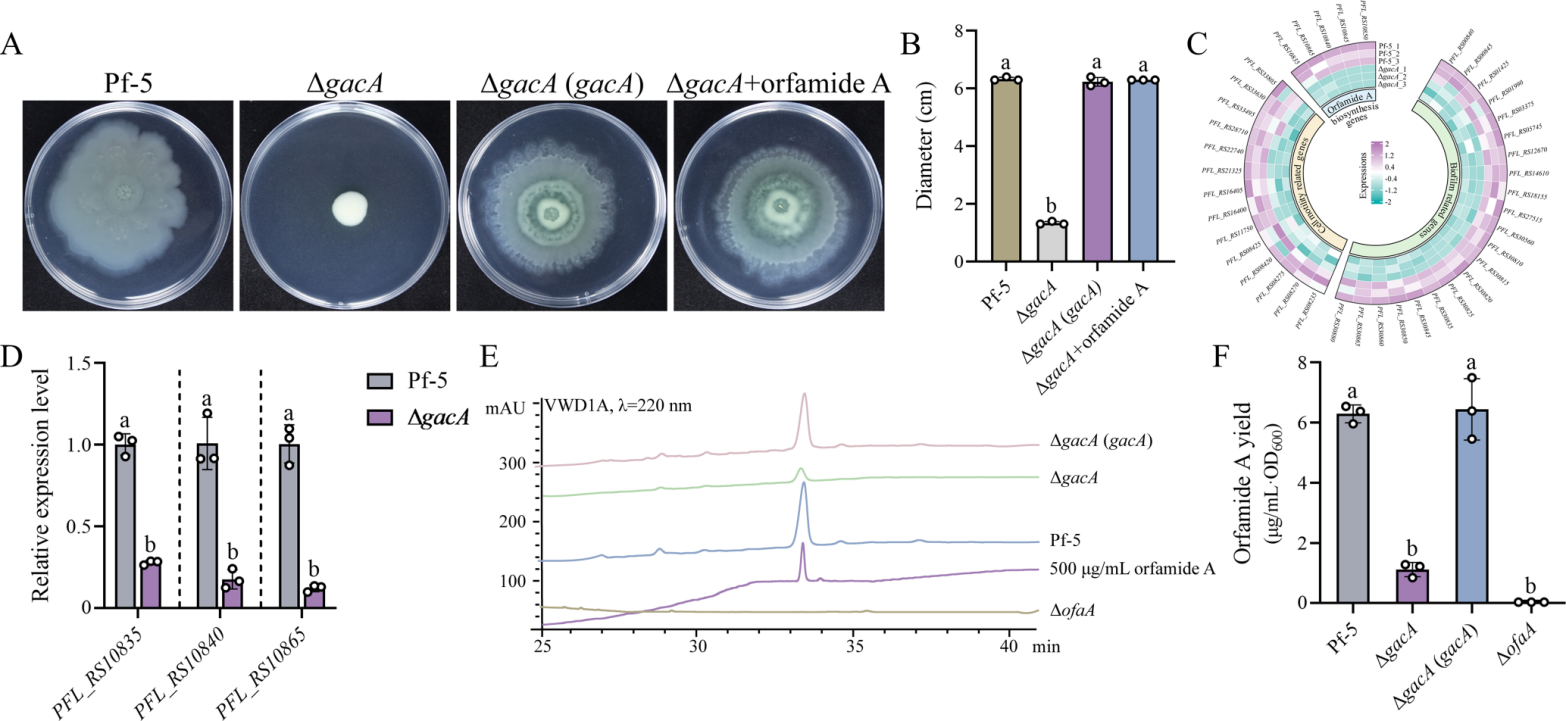
GacS/A governs swarming motility and orfamide A production in Pf-5. (**A** and **B**) Swarming motility was tested in semi-solid KB medium containing 0.7% agar (A) and the colony diameter was measured (B). Δ*gacA *+ orfamide A, swarming motility of Δ*gacA* on orfamide A-supplemented (5 μg/ml) plates. All the plate experiments shown in panel (A) were representative of three separate experiments with similar results. Data in panel (B) were presented as the mean ± SD from three independent experiments (*n* = 3) with similar results. Different lowercase letters within the same row indicate statistically significant differences. (**C**) The representative genes expression architecture in the transcriptomic data of Pf-5 and Δ*gacA*. (**D**) Validation of RNA-seq results via qRT-PCR. Data were analyzed by Student’s *t*-test, and differences were considered significant at *P* < 0.05. Different lowercase letters within the same row indicate statistically significant differences. (**E** and **F**) HPLC analysis was performed to check orfamide A synthesis in Pf-5, Δ*gacA*, and Δ*gacA* (*gacA*) (E), and the yield was quantified and normalized to cell density (F). All HPLC results in panel (E) were repeated for three times separately with similar results. Data in panel (F) were presented as the mean ± SD from three independent experiments (*n* = 3) with similar results. Different lowercase letters within the same row indicate statistically significant differences.

The GacS/A two-component system regulates a wide range of physiological processes in *Pseudomonas* strains [9, 11]. In this study, when the *gacA* gene of *P. protegens* Pf-5 was knocked out, a remarkable reduction in swarming motility was observed in the Δ*gacA* mutant, while the phenotype was fully restored in the complemented strain Δ*gacA* (*gacA*) (Fig. 1A and B). This result confirmed that GacA is required for normal swarming motility in Pf-5.

To identify the factors underlying this motility defect, RNA sequencing (RNA-seq)-based transcriptomic analysis was performed. Total RNAs of Pf-5 and Δ*gacA* were extracted for Illumina HiSeq sequencing. As shown in Supplementary Fig. S1A, there were 982 differentially expressed genes (DEGs, |log_2_(Fold Change)| >1, FDR < 0.05) between the wild-type Pf-5 and the Δ*gacA* mutant, including 467 upregulated and 515 downregulated genes. Among the downregulated genes were those associated with swarming motility (e.g. *PFL_RS22740, PFL_RS11750, PFL_RS08425, PFL_RS16405, PFL_RS21325*) and biofilm formation (e.g. *PFL_RS00845, PFL_RS14610, PFL_RS18155, PFL_RS12670*). Notably, the entire orfamide A biosynthetic cluster (e.g. *PFL_RS10840-10850, PFL_RS10835, PFL_RS10865*) was significantly downregulated in the Δ*gacA* mutant, suggesting that reduced orfamide A production may contribute to the observed motility defect (Fig. 1C). The downregulation of selected genes was validated by qRT-PCR (Fig. 1D), confirming the reliability of the RNA-seq data.

To determine whether the orfamide A production indeed changed at the metabolic level, we quantified orfamide A yields by HPLC. Consistent with the transcriptomic data, orfamide A production was drastically reduced in the Δ*gacA* mutant, similar to the Δ*ofaA* mutant lacking the orfamide A biosynthesis gene, but was completely restored in the complemented strain Δ*gacA* (*gacA*) (Fig. 1E and F). Moreover, exogenous addition of orfamide A (5 μg/ml) rescued the swarming defect of Δ*gacA* mutant, confirming that GacS/GacA regulates swarming motility in Pf-5 by modulating the production of orfamide A (Fig. 1A and B).

### The GacS/A two-component system activates transcription of RsmX, RsmY, and RsmZ

**Figure 2.**
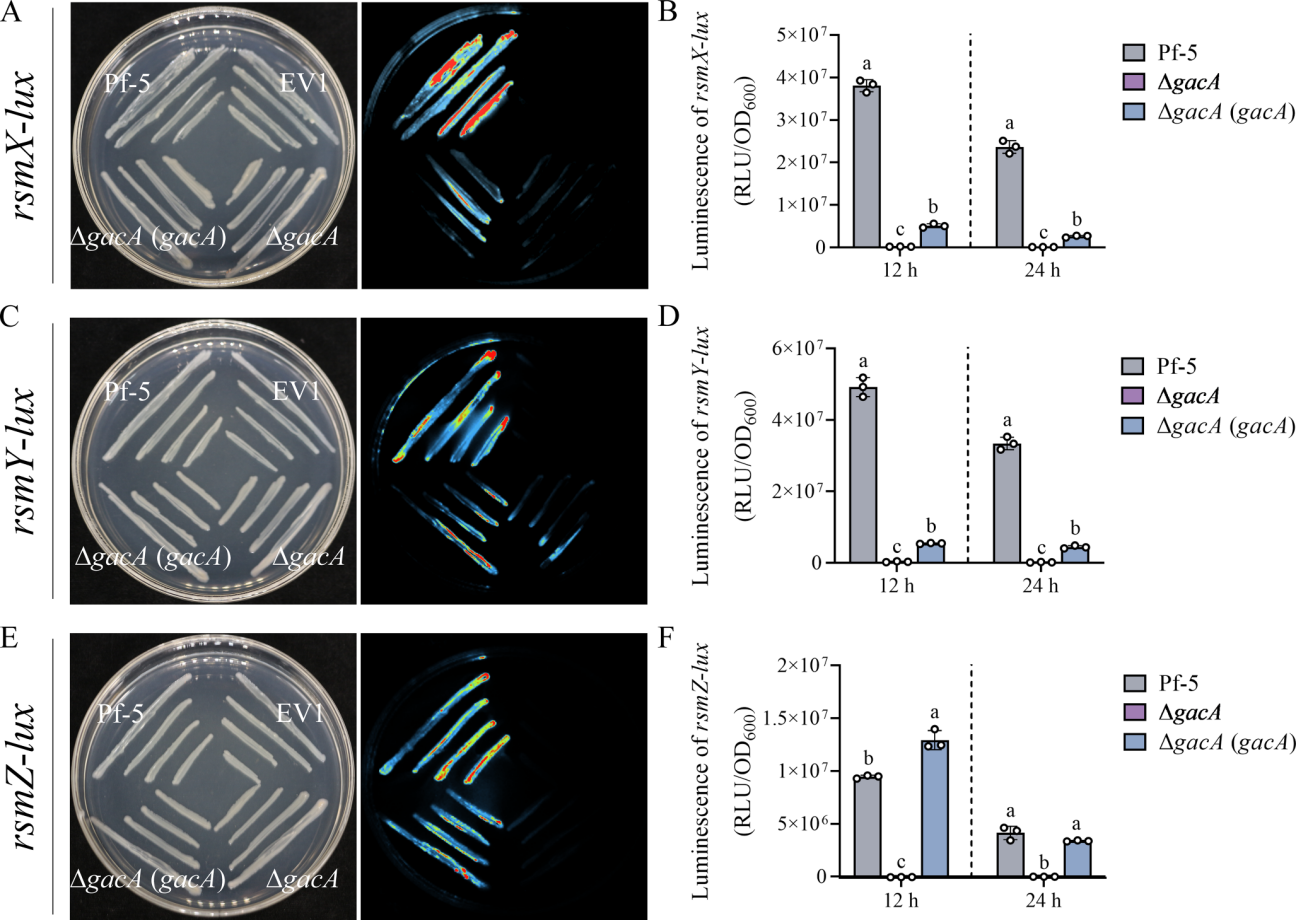
GacS/A activates the transcription of *rsmX, rsmY*, and *rsmZ*. (**A, C**, and **E**) The transcription of three sRNAs were directly indicated by the luciferase dependent luminescence intensity in Pf-5, Δ*gacA*, and Δ*gacA* (*gacA*) strains hosting *the transcriptional P_rsmX_::lux* (A), *P_rsmY_::lux* (C), and *P_rsmZ_::lux* (E) *fusion reporters* after 24 h culture. EV, empty vector. All the plate experiments shown in A, C, and E were representative of three separate experiments with similar results. (**B, D**, and **F**) To estimate the luciferase luminescence activity, samples taken after 12 and 24 h were lysed and estimated by multifunctional microplate detector. Data in B, D, and F were presented as the mean ± SD from three independent experiments (*n* = 3) with similar results. Different lowercase letters within the same row indicate statistically significant differences.

The GacS/A two-component system activates transcription of small RNAs in *Pseudomonas* strains, which typically function as key regulators in the Gac/Rsm signal transduction pathway [9]. Here, three sRNAs (RsmX, RsmY, and RsmZ) were exist in Pf-5 and selected for further analysis. The putative promoter region of *rsmX, rsmY*, and *rsmZ* were cloned into the pME6522-*lux* reporter plasmids, which was then introduced into Pf-5, Δ*gacA*, and Δ*gacA* (*gacA*) strains to establish the reporter system. Compared to the wild-type strain Pf-5, the expression levels of *rsmX*-*lux, rsmY*-*lux*, and *rsmZ*-*lux* were significantly decreased in ∆*gacA* but restored to wild-type level in the complemented Δ*gacA* (*gacA*) strain (Fig. 2). These results indicated that GacS/A activates the transcription of *rsmX, rsmY*, and *rsmZ*.

### sRNAs synergistically regulate the swarming motility and orfamide A production

**Figure 3.**
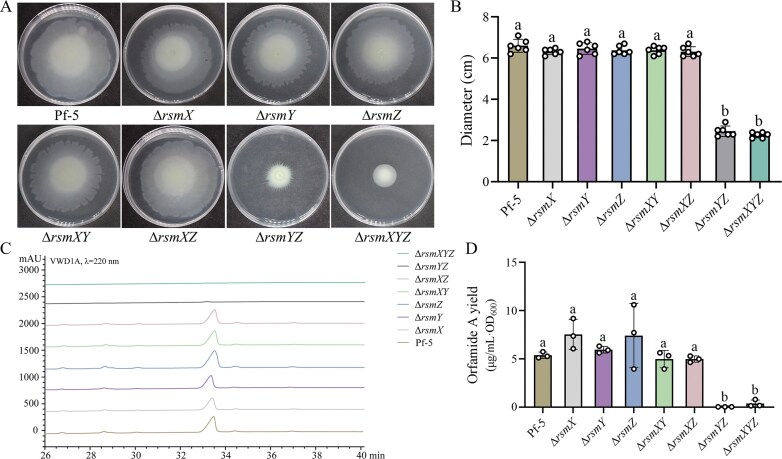
sRNAs positively regulate swarming motility and the production of orfamide A in a synergistic manner. (**A** and **B**) Swarming motility of Pf-5, Δ*rsmX*, Δ*rsmY*, Δ*rsmZ*, Δ*rsmXY*, Δ*rsmXZ*, Δ*rsmYZ*, and Δ*rsmXYZ* was tested in semi-solid KB medium containing 0.7% agar (A) and the colony diameter was measured (B). All the plate experiments shown in panel (A) were representative of six separate experiments with similar results. Data in panel (B) were presented as the mean ± SD from six independent experiments (*n* = 6) with similar results. Different lowercase letters within the same row indicate statistically significant differences. (**C** and **D**) HPLC analysis was performed to check orfamide A synthesis in these strains (C), and the yield was quantified and normalized to cell density (D). All HPLC in panel (C) were repeated for three times separately with similar results. Data in panel (D) were presented as the mean ± SD from three separate experiments (*n* = 3) with similar results; mean ± SD. Different lowercase letters within the same row indicate statistically significant differences.

In order to investigate the specific impact of RsmX, RsmY, and RsmZ on swarming motility and orfamide A production, single mutants (Δ*rsmX*, Δ*rsmY*, and Δ*rsmZ*), double mutants (Δ*rsmXY*, Δ*rsmXZ*, and Δ*rsmYZ*), and triple mutant (Δ*rsmXYZ*) were constructed. The results showed that single deletion mutants of Δ*rsmX*, Δ*rsmY* and Δ*rsmZ* had no significant effect on swarming motility (Fig. 3A and B) or orfamide A production (Fig. 3C and D) compared to the wild-type Pf-5. In contrast, both swarming motility and orfamide A yield were markedly reduced in the Δ*rsmYZ* and Δ*rsmXYZ* mutants. These results suggested that the three sRNAs function synergistically in regulating these phenotypes. Notably, the Δ*rsmXY* and Δ*rsmXZ* mutants retained wild-type levels of motility and orfamide A production, implying that RsmY and RsmZ play dominant roles, while RsmX plays a minor role in the regulation of orfamide A biosynthesis.

### A detailed dissection of Rsm-dependent regulation of orfamide A biosynthesis

**Figure 4.**
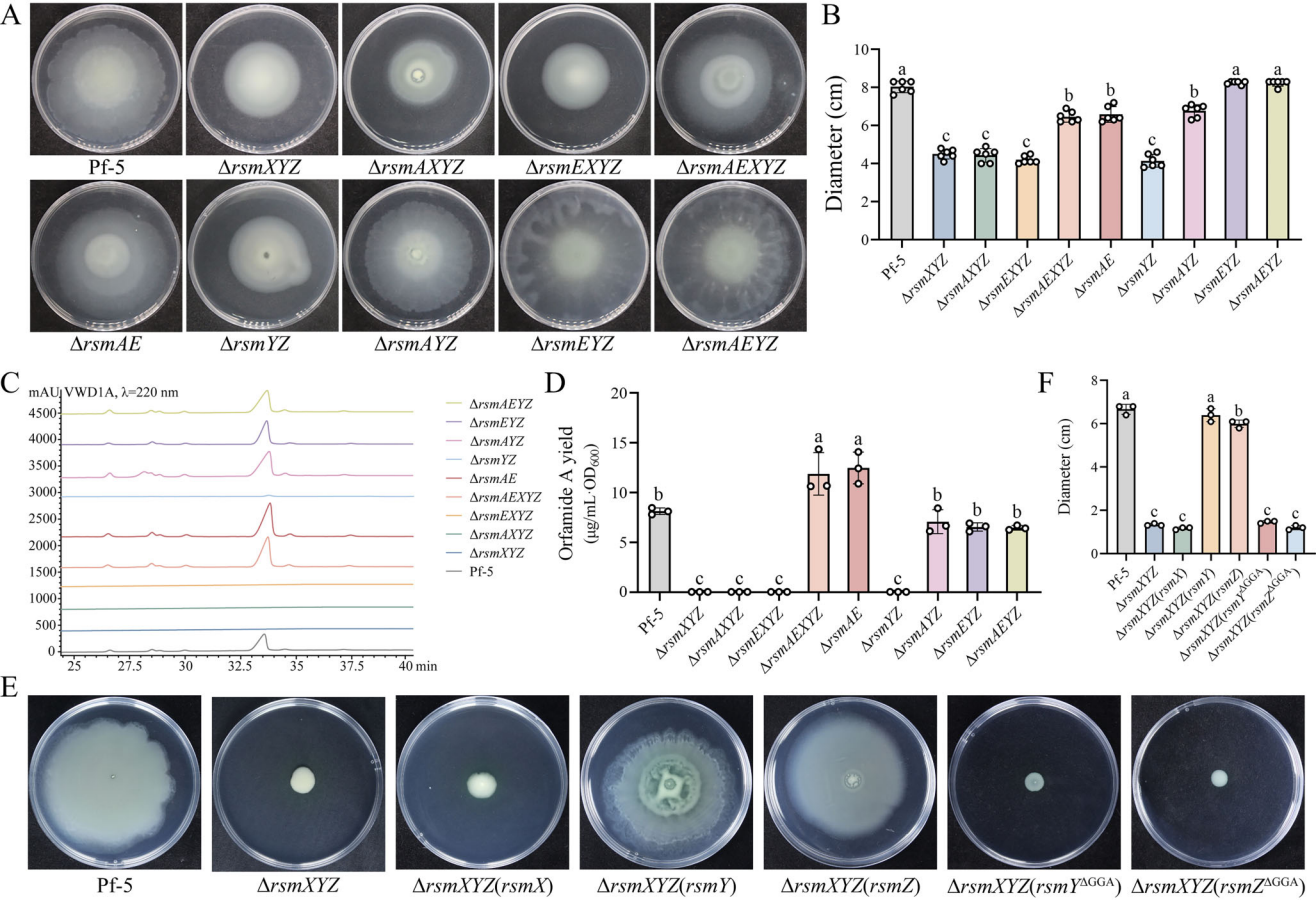
The members of the Rsm system regulate gradually swarming motility and the production of orfamide A. (**A** and **B**) Swarming motility of Pf-5, Δ*rsmXYZ*, Δ*rsmAXYZ*, Δ*rsmEXYZ*, Δ*rsmAEXYZ*, Δ*rsmAE*, Δ*rsmYZ*, Δ*rsmAYZ*, Δ*rsmEYZ*, and Δ*rsmAEYZ* was tested (A) and the colony diameter was measured (B). All the plate experiments shown in panel (A) were representative of six separate experiments with similar results. Data in panel (B) were presented as the mean ± SD from six independent experiments (*n* = 6) with similar results. Different lowercase letters within the same row indicate statistically significant differences. (**C** and **D**) HPLC analysis was performed to check orfamide A synthesis in these strains (C), and the yield was quantified and normalized to cell density (D). All HPLC in panel (C) were repeated for at least three times separately with similar results. Data in panel (D) were presented as the mean ± SD from three separate experiments (*n* = 3) with similar results. Different lowercase letters within the same row indicate statistically significant differences. (**E** and **F**) Swarming motility of Pf-5, Δ*rsmXYZ*, Δ*rsmXYZ* (*rsmX*), Δ*rsmXYZ* (*rsmY*), Δ*rsmXYZ* (*rsmZ*), Δ*rsmXYZ* (*rsmY*^ΔGGA^), and Δ*rsmXYZ* (*rsmZ*^ΔGGA^) was tested (E) and the colony diameter was measured (F). All the plate experiments shown in panel (E) were representative of three separate experiments with similar results. Data in panel (F) were presented as the mean ± SD from three independent experiments (*n* = 3) with similar results. Different lowercase letters within the same row indicate statistically significant differences.

RsmA/CsrA-family repressor proteins suppress translation of target mRNAs and sRNAs can sequester these repressors [2, 11, 21]. The two repressor proteins, RsmA and RsmE, belonging to the RsmA/CsrA family, are present in Pf-5. To elucidate whether RsmA and RsmE negatively regulate the biosyntesis of orfamide A, quadruple mutants (Δ*rsmAXYZ* and Δ*rsmEXYZ*) and quintuple mutant (Δ*rsmAEXYZ*) were generated. Both quadruple mutants exhibited significantly reduced swarming motility and orfamide A production, resembling the Δ*rsmXYZ* phenotype (Fig. 4A–D). Strikingly, when both repressor genes (*rsmA* and *rsmE*) were deleted, as in Δ*rsmAE* and Δ*rsmAEXYZ*, both swarming motility and orfamide A yield were restored to wild-type levels. These findings indicated that either RsmA or RsmE alone is sufficient to suppress orfamide A biosynthesis, highlighting their functional redundancy.

To further investigate the specific contribution of each sRNA, we constructed complementation strains Δ*rsmXYZ* (*rsmX*), Δ*rsmXYZ* (*rsmY*), and Δ*rsmXYZ* (*rsmZ*). Swarming motility assays showed that introduction of either *rsmY* or *rsmZ* completely restored motility to wild-type levels. In contrast, introduction of *rsmX* failed to rescue the Δ*rsmXYZ* phenotype, indicating that RsmX alone is insufficient to overcome the loss of all three sRNAs (Fig. 4E and F).

Previous studies showed that the Rsm proteins exert their regulatory function by binding to a *cis*-element GGA located in the 5′UTR regions of target mRNAs [36–38]; To determine whether this motif is required for RsmY- and RsmZ-mediated regulation, we deleted the GGA element and generated mutants Δ*rsmXYZ* (*rsmY*^ΔGGA^) and Δ*rsmXYZ* (*rsmZ*^ΔGGA^). The resulting mutants exhibited significantly reduced swarming motility compared to their GGA-intact counterparts (Fig. 4E and F), confirming that the GGA motif is essential for RsmY and RsmZ function.

We next asked whether RsmX can function in the absence of one or both repressor proteins. To address this, mutants lacking combinations of *rsmY, rsmZ*, and one or both repressors were constructed (Δ*rsmAYZ*, Δ*rsmEYZ*, and Δ*rsmAEYZ*). In contrast to the Δ*rsmYZ* mutant, which exhibits severe defects in both swarming motility and orfamide A production, all three repressor-deficient mutants displayed phenotypes comparable to wild-type Pf-5 (Fig. 4A and B). HPLC analysis confirmed that orfamide A levels in these strains were restored accordingly (Fig. 4C and D). Taken together, these results demonstrated that RsmY and RsmZ function as the primary sRNAs mediating derepression of orfamide A biosynthesis, whereas RsmX plays a compensatory role in the absence of RsmY and RsmZ, functioning to alleviate the repression imposed by either RsmA or RsmE.

### The Rsm system mediates the expression of *luxR1, luxR2*, and the *ofa* operon

**Figure 5.**
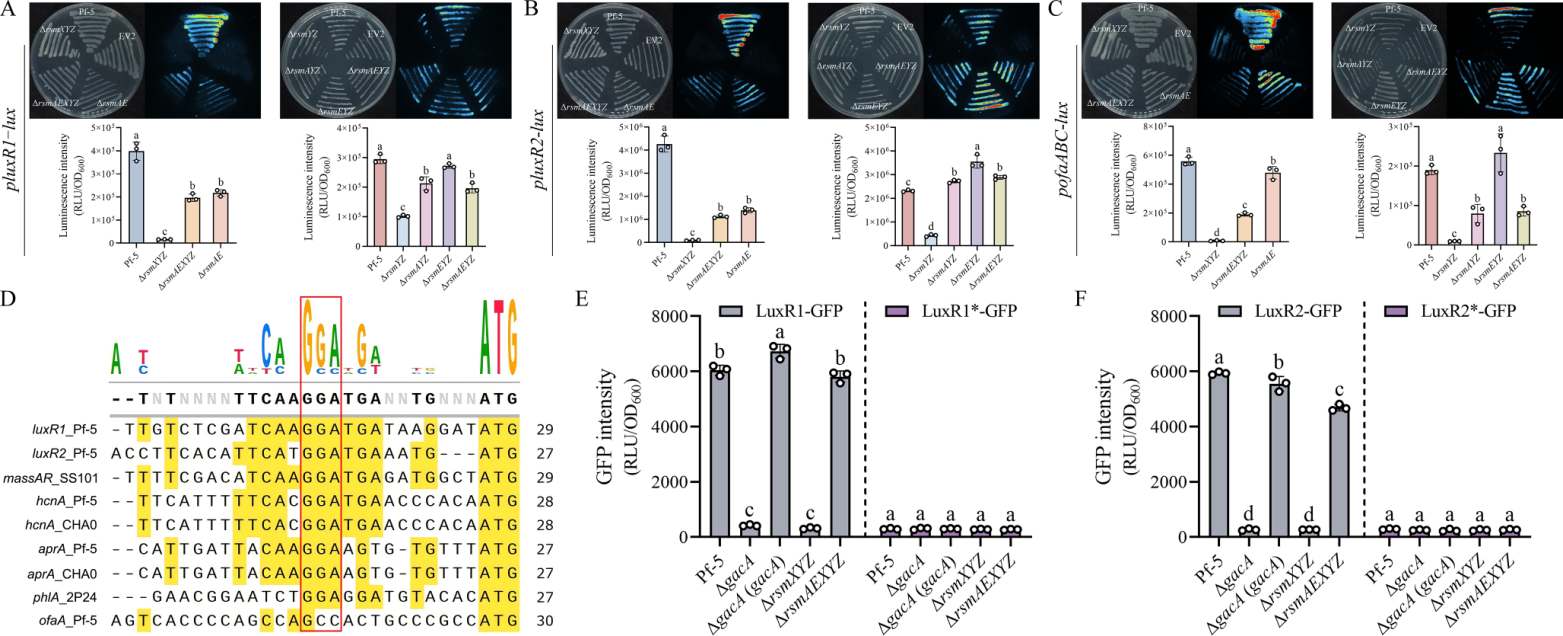
(**A–C**) Luciferase activity of *luxR1, luxR2* and *ofaABC* promoters in different strains using the transcriptional *P_luxR1_::lux* (A), *P_luxR2_::lux* (B), and *P_ofaABC_::lux* (C) fusion reporters. The upper figures represented the luciferase intensity of these strains growing on agar plates at 24 h. EV, empty vector. All the plate experiments shown were representative of three separate experiments with similar results. The column diagrams represented the quantitative results of the luciferase activity after 24 h culture. Data were presented as the mean ± SD from three separate experiments (*n* = 3) with similar results. Different lowercase letters within the same row indicate statistically significant differences. (**D**) Sequence alignment of 5′UTR of genes related to Rsm proteins interaction from different strains. GGA is the relatively conserve motif of these genes. (**E** and **F**) GFP intensity of *luxR1* and *luxR2* promoters in different strains at 12 h using the translational *P_luxR1_::gfp* (E) and *P_luxR2_::gfp* (F) fusions. LuxR1*, the GGA motif of *luxR1* has been converted to AGA. LuxR2*, the GGA motif of *luxR2* has been converted to CCA. Data were presented as the mean ± SD from three independent experiments (*n* = 3) with similar results. Different lowercase letters within the same row indicate statistically significant differences.

According to the RNA-seq results, two downregulated genes (*PFL_RS10835, PFL_RS10865*) in Δ*gacA* mutant attributed out attention. These LuxR-like transcription factors encoding genes, designated as *luxR1* and *luxR2*, located upstream and downstream of the *ofa* operon (*PFL_RS10840-10850*) in the genome of Pf-5 (Supplementary Fig. S2A). To investigate whether the expression of *luxR1, luxR2*, and *ofa* operon was influenced by the Rsm system, the promoter region of these genes was cloned into the pME6015-*lux* and subsequently transformed into the corresponding strains. Luciferase luminescence activity analysis showed that the expression of *luxR1*-*lux, luxR2*-*lux*, and *ofa*-*lux* was significantly downregulated in the Δ*rsmYZ* and Δ*rsmXYZ* mutants (Fig. 5A–C). In contrast, these reporter activities were significantly upregulated, to varying extents, in Δ*rsmAYZ*, Δ*rsmEYZ*, Δ*rsmAEYZ*, Δ*rsmAEXYZ*, and Δ*rsmAE* mutants compared with Δ*rsmYZ* and Δ*rsmXYZ*. These results suggest that *luxR1, luxR2*, and the *ofa* operon may be targets of Gac–Rsm cascade.

### LuxR1 and LuxR2 are direct targets of the Rsm system

The Rsm proteins exert post-transcriptional control by binding to a conserved *cis*-element, GGA [36, 38]. According to the multiple EM for motif elicitation (MEME) analysis, both *luxR1* and *luxR2* harbor this Rsm binding *cis*-element, whereas the *ofa* operon is devoid of it (Fig. 5D). Therefore, we used a GFP reporter assay to assess whether RsmA and RsmE exert translational repression on *luxR1* and *luxR2* expression.

The translational fusions of *luxR1* and *luxR2* with *gfp* were transformed into relevant strains and the GFP intensity showed that the expression of both *luxR1* and *luxR2* was significantly reduced in the Δ*gacA* and Δ*rsmXYZ* mutants compared to wild-type Pf-5 at both 12 and 24 h time points (Fig. 5E and F, and Supplementary Fig. S2B and C). Importantly, expression was restored to wild-type levels in the Δ*gacA* (*gacA*) and Δ*rsmAEXYZ* mutants, indicating that RsmA and RsmE are responsible for repressing the expression of *luxR1* and *luxR2*.

To determine whether this repression depends on the predicted GGA motifs, we introduced point mutations in the *luxR1* and *luxR2* 5′UTRs, converting GGA to AGA and CCA, respectively. Interestingly, there was no difference in GFP intensity among the wild-type Pf-5, Δ*gacA*, Δ*gacA* (*gacA*), Δ*rsmXYZ*, and Δ*rsmAEXYZ*, indicating that GGA motif is necessary for Rsm-mediated repression of *luxR1* and *luxR2* (Fig. 5E and F).

**Figure 6.**
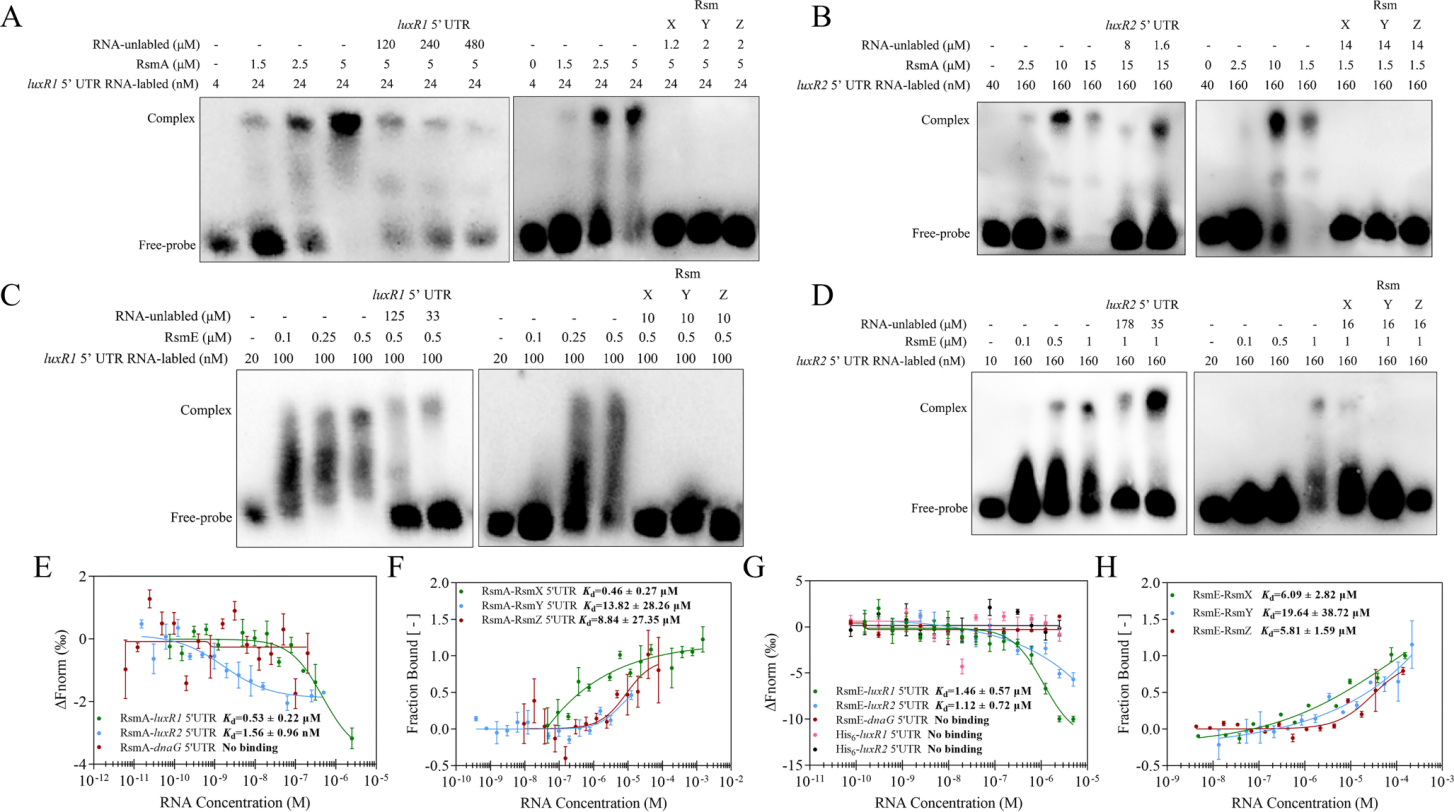
The Rsm system directly acts on LuxR1 and LuxR2 determined by RNA–EMSA and MST assays. (**A**) The RNA–EMSA experiments showed that RsmA protein interacted with biotin-labeled *luxR1* 5′UTR RNA (left), and the unlabeled probes, RsmX, RsmY, RsmZ, attenuated the interaction by competing with *luxR1* 5′UTR (right). (**B**) RNA–EMSA experiments showed that RsmA protein interacted with biotin-labeled *luxR2* 5′UTR RNA (left), and the interaction was diminished by unlabeled RsmX, RsmY, and RsmZ (right). (**C** and **D**) RNA–EMSA showed that RsmE protein interacted with biotin-labeled *luxR1* 5′UTR (C, left) and *luxR2* 5′UTR (D, left), and sRNAs attenuated this interaction (C and D, right). All blots shown were representative of three separate experiments with similar results. (**E** and **F**) MST assays showed that RsmA protein bound to *luxR1* 5′UTR (E), *luxR2* 5′UTR (E), RsmX (F), RsmY (F), and RsmZ (F). The *dnaG* 5′UTR was included as the negative RNA control. (**G** and **H**) MST assays showed that RsmE protein bound to *luxR1* 5′UTR (G), *luxR2* 5′UTR (G), RsmX (H), RsmY (H), and RsmZ (H). The *dnaG* 5′UTR was included as the negative RNA control, and His_6_ protein was included as the negative protein control. All MST assays were presented as the mean ± SD from three independent experiments (*n* = 3) with similar results.

The interaction between the two transcription factors and the Rsm system was further investigated *in vitro*. The results of RNA–EMSA showed that the RsmA protein formed clear bands, indicating protein–RNA complexes, with the *luxR1* and *luxR2* 5′UTR RNA probes. Addition of unlabeled RNA fragments abolished the shifts in a concentration-dependent manner, confirming specific binding (Fig. 6A and B). These results indicated that RsmA directly binds to the *luxR1* and *luxR2* 5′UTR regions, thereby inhibiting the expression of the two transcription factors. Furthermore, we observed that the clear bands diminished upon addition of sRNAs (RsmX, RsmY, and RsmZ), suggesting that sRNAs relieve repression of *luxR1* and *luxR2* by competing for RsmA binding (Fig. 6A and B). Similar results were obtained for RsmE, which also formed retarded bands with the *luxR1* and *luxR2* 5′UTR probes; these bands were progressively diminished upon addition of unlabeled RNA competitors (Fig. 6C and D). Additionally, substitution of the Rsm-binding motif of *luxR1* 5′UTR (GGA to AGA) and *luxR2* 5′UTR (GGA to CCA) abolished the binding of RsmA and RsmE with *luxR1* and *luxR2* 5′UTR (Supplementary Fig. S3A–D).

As expected, MST analysis revealed that 5′UTR of *luxR1* and *luxR2* bound to the RsmA protein with dissociation constants (*K*_d_) of 0.53 ± 0.22 μM and 1.56 ± 0.96 nM, respectively (Fig. 6E). RsmX, RsmY, and RsmZ were also found to interact with the RsmA protein with affinity *K*_d_ values of 0.46 ± 0.27 μM, 13.82 ± 28.26 μM, and 8.84 ± 27.35 μM, respectively (Fig. 6F). Similarly, the RsmE protein bound to *luxR1, luxR2* 5′UTR RNA, RsmX, RsmY, and RsmZ, with *K*_d_ of 1.46 ± 0.57 μM,1.12 ± 0.72 μM, 6.09 ± 2.82 μM, 19.64 ± 38.72 μM, and 5.81 ± 1.59 μM, respectively (Fig. 6G and H).

Additionally, given that the *ofa* operon lacks a GGA *cis*-element, EMSA and MST analysis confirmed that RsmA and RsmE do not bind to the *ofa* 5′UTR (Supplementary Fig. S3F and G). Collectively, these findings established LuxR1 and LuxR2 as direct targets of RsmA and RsmE, placing them downstream of the Gac-Rsm cascade and near the orfamide A biosynthetic gene cluster.

### LuxR2 activates the expression of LuxR1, which directly regulates orfamide A production

**Figure 7.**
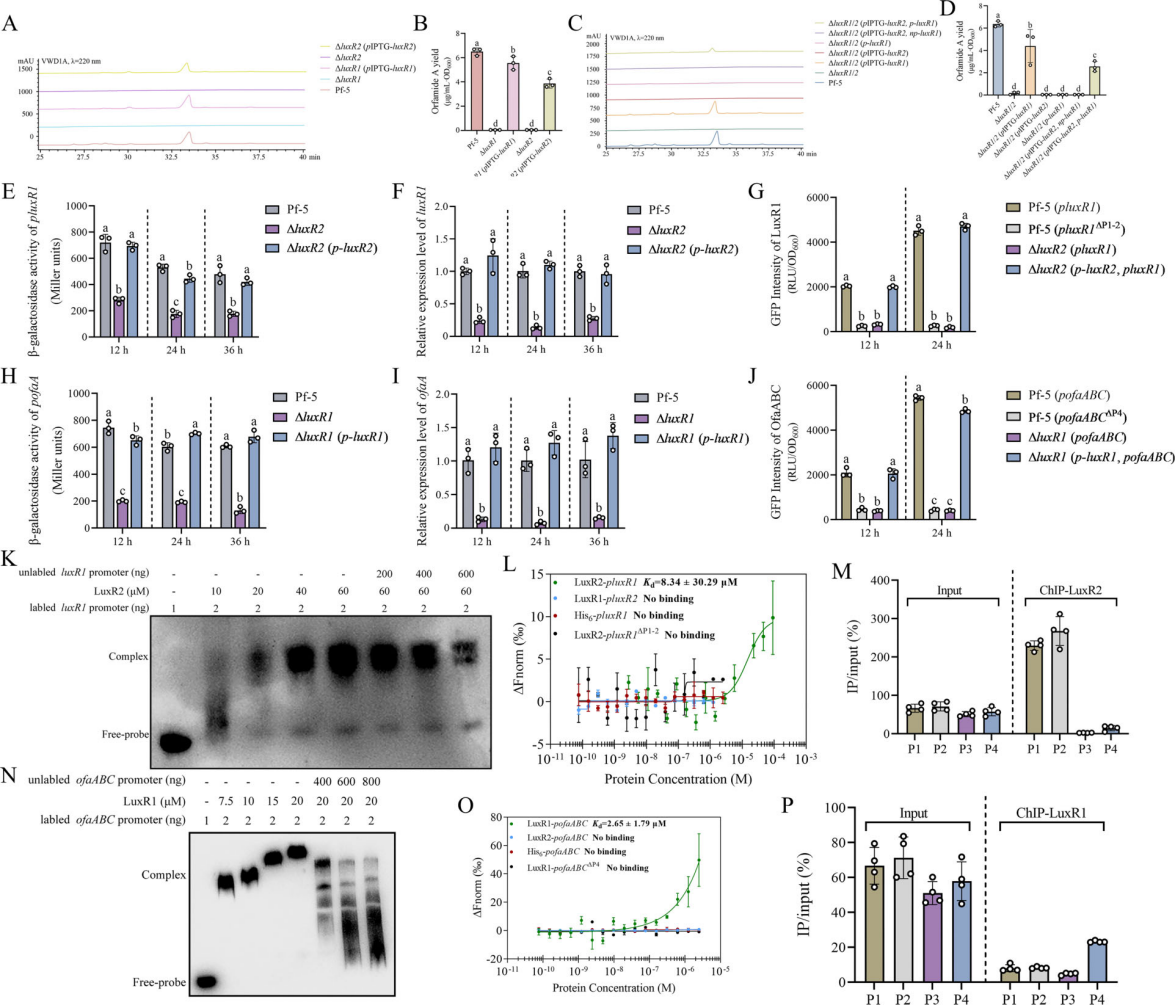
LuxR1 and LuxR2 govern the production of orfamide A. (**A** and **B**) The production of orfamide A in Pf-5, Δ*luxR1*, Δ*luxR1* (*p*IPTG-*luxR1*), Δ*luxR2*, and Δ*luxR2* (*p*IPTG*-luxR2*). HPLC results were shown in panel (A), and the column diagram (B) showed the calculated orfamide A yield. Δ*luxR1* (*p*IPTG-*luxR1*), Δ*luxR1* carrying the pME6032-*luxR1* plasmid. Δ*luxR2* (*p*IPTG-*luxR2*), Δ*luxR2* carrying the pME6032-*luxR2* plasmid. (**C** and **D**) The production of orfamide A in Δ*luxR1*/*2* related strains: Δ*luxR1*/*2*, Δ*luxR1*/*2* (*p*IPTG-*luxR1*), Δ*luxR1*/*2* (*p*IPTG-*luxR2*), Δ*luxR1*/*2* (*p*-*luxR1*), Δ*luxR1*/*2* (*p*IPTG*-luxR2, np*-*luxR1*), and Δ*luxR1*/*2* (*p*IPTG*-luxR2, p*-*luxR1*). HPLC (C) and calculated orfamide A yields (D) were shown. Δ*luxR1*/*2* (*p*IPTG-*luxR1*), Δ*luxR1*/*2* carrying the pME6032-*luxR1* plasmid. Δ*luxR1*/*2* (*p*IPTG-*luxR2*), Δ*luxR1*/*2* carrying the pME6032-*luxR2* plasmid. Δ*luxR1*/*2* (*p*-*luxR1*), Δ*luxR1*/*2* carrying the pBBR1MCS-5-*p*-*luxR1* plasmid, in which *p* represents the native promoter of *luxR1*. Δ*luxR1*/*2* (*p*IPTG*-luxR2, np*-*luxR1*), Δ*luxR1*/*2* carrying the pME6032-*luxR2* and pBBR1MCS-5-*np*-*luxR1* plasmids, in which *np* represents no native promoter of *luxR1*. Δ*luxR1*/*2* (*p*IPTG*-luxR2, p*-*luxR1*), Δ*luxR1*/*2* carrying the pME6032-*luxR2* and pBBR1MCS-5-*p*-*luxR1* plasmids. All HPLC in panels (A) and (C) were repeated for three times separately with similar results. Data in panel (B) and (D) were quantified and normalized to cell density, and were presented as the mean ± SD from three independent experiments (*n* = 3) with similar results. Different lowercase letters within the same row indicate statistically significant differences. (**E**) β-Galactosidase activity of *luxR1* promoter activity using the transcriptional *P_luxR1_::lacZ* fusion reporter expressed in Pf-5, Δ*luxR2* and Δ*luxR2* (*p-luxR2*) at 12, 24, and 36 h. (**F**) qRT-PCR analysis of *luxR1* mRNA levels in Pf-5, Δ*luxR2* and Δ*luxR2* (*p-luxR2*) at 12, 24, and 36 h. (**G**) GFP intensity of *luxR1* promoter using the translational *P_luxR1_::gfp* fusion in Pf-5, Δ*luxR2* and Δ*luxR2* (*p-luxR2*) at 12 and 24 h. *pluxR1*^ΔP1-2^, the *luxR1* promoter lacking the P1 and P2 regions. Δ*luxR2* (*p-luxR2*) in panels (E–G), Δ*luxR2* carrying the pBBR1MCS-5-*p*-*luxR2* plasmid, in which *p* represents the native promoter of *luxR2*. (**H**) β-Galactosidase activity of *ofaABC* promoter activity using the transcriptional *P_ofaABC_::lacZ* fusion reporter expressed in Pf-5, Δ*luxR1* and Δ*luxR1* (*p-luxR1*) at 12, 24, and 36 h. (**I**) qRT-PCR analysis of *ofaA* mRNA levels in Pf-5, Δ*luxR1* and Δ*luxR1* (*p-luxR1*) at 12, 24, and 36 h. (**J**) GFP intensity of *ofaABC* promoter using the translational *P_ofaABC_::gfp* fusion in Pf-5, Δ*luxR1* and Δ*luxR1* (*p-luxR1*) at 12 and 24 h. *pofaABC*^ΔP4^, the *ofaABC* promoter lacking the P4 region. Δ*luxR1* (*p-luxR1*) in panels (H–J), Δ*luxR1* carrying the pBBR1MCS-5-*p*-*luxR1* plasmid. Data in panels (E–J) were presented as the mean ± SD from three independent experiments (*n* = 3) with similar results. Different lowercase letters within the same row indicate statistically significant differences. (**K**) The DNA–EMSA experiment showed that the LuxR2 protein interacted with and *luxR1* promoter. (**L**) MST assays showed that LuxR2 protein bound to the *luxR1* promoter. *pluxR1*^ΔP1-2^, the *luxR1* promoter lacking the P1 and P2 regions. His_6_ protein was included as the negative protein control. The binding of LuxR1 protein and *luxR2* promoter was also included as the negative control. (**M**) The enrichment region of GFP-LuxR2 at *luxR1* promoter detected by ChIP-qPCR. (**N**) DNA–EMSA experiment showed that LuxR1 protein interacted with the *ofaABC* promoter. (**O**) MST assays showed that LuxR1 protein bound to the *ofaABC* promoter. *pofaABC*^ΔP4^, the *ofaABC* promoter lacking the P4 region. His_6_ protein was included as the negative protein control. The binding of LuxR2 protein and *ofaABC* promoter was also included as the negative control. (**P**) The enrichment region of GFP–LuxR1 at *ofaABC* promoter detected by ChIP-qPCR. Blots shown in panels (K) and (N) are representative of three separate experiments with similar results. MST assays in L and O were presented as the mean ± SD from three independent experiments (*n* = 3) with similar results. All ChIP- and input-DNA samples in panels (M) and (P) were quantified by quantitative PCR assays with the primer pair indicated in Supplementary Table S2, and data in panels (M) and (P) were presented as the mean ± SD from three independent experiments (*n* = 3) with similar results.

To elucidate the regulatory roles of LuxR1 and LuxR2 in orfamide A biosynthesis, single and double mutants (∆*luxR1*, ∆*luxR2*, and ∆*luxR1*/*2*) were constructed. By contrast to the wild-type Pf-5, neither Δ*luxR1* nor Δ*luxR2* produced orfamide A, indicating that both LuxR1 and LuxR2 are required for its biosynthesis (Fig. 7A and B). To gain deeper insights into the regulatory pathway of orfamide A synthesis, the native promoters of *luxR1* and *luxR2* were replaced with IPTG-inducing promoters, and these constructs were introduced into the double mutant Δ*luxR1*/*2* to generate the mutants Δ*luxR1*/*2* (*p*IPTG-*luxR1*) and Δ*luxR1*/*2* (*p*IPTG*-luxR2*). The orfamide A yields of these two mutants was estimated, and the results showed that only Δ*luxR1*/*2* (*p*IPTG*-luxR1*) produced orfamide A upon induction with 1 mM IPTG (Fig. 7C and D), suggesting that LuxR1 directly mediates orfamide A biosynthesis. Notably, Δ*luxR1*/*2* (*p*-*luxR1*), carrying *luxR1* under its native promoter, failed to produce orfamide A, implying that *luxR2* is required to activate *luxR1* transcription. To verify this hypothesis, the two mutants, Δ*luxR1*/*2* (*p*IPTG*-luxR2, np*-*luxR1*) and Δ*luxR1*/*2* (*p*IPTG*-luxR2, p*-*luxR1*), were constructed. Upon IPTG induction, Δ*luxR1*/*2* (*p*IPTG-*luxR2, p*-*luxR1*) produced orfamide A, whereas Δ*luxR1*/*2* (*p*IPTG-*luxR2, np*-*luxR1*) did not (Fig. 7C and D), confirming that LuxR2 acts upstream to activate *luxR1*, which in turn directly regulates the *ofa* operon.

Expression analysis further supported this hierarchy. β-Galactosidase assays, qRT-PCR and GFP intensity showed that *luxR1* expression was markedly reduced in Δ*luxR2* compared with wild-type Pf-5, but restored in Δ*luxR2* (*p*-*luxR2*) (Fig. 7E–G). Similarly, expression of the *ofa* operon was reduced in Δ*luxR1* and restored in Δ*luxR1* (*p*-*luxR1*) (Fig. 7H–J). In contrast, the *luxR2* expression was comparable in Pf-5 and Δ*luxR1* (Supplementary Fig. S4A), indicating that LuxR1 does not regulate *luxR2* transcription. To confirm the interactions between LuxR1, LuxR2, and the *ofa* operon promoter *in vitro*, DNA-EMSA and MST experiments were performed. The results showed that the LuxR2 protein formed retarded bands with the labeled *luxR1* promoter, and which was diminished upon the addition of the unlabeled *luxR1* probe (Fig. 7K). Consistently, LuxR1 protein also formed retarded bands with the *ofaABC* promoter (Fig. 7N). MST experiments further demonstrated that the LuxR2 protein bound to the *luxR1* promoter with a *K*_d_ value of 8.34 ± 30.29 μM (Fig. 7L), and LuxR1 protein bound to the *ofaABC* promoter with a *K*_d_ value of 2.65 ± 1.79 μM (Fig. 7O), while there was no binding between LuxR1 protein with *luxR2* promoter, and LuxR2 protein with *ofaABC* promoter (Fig. 7L and O, and Supplementary Fig. S4B–D). ChIP-qPCR assays were subsequently performed to map the binding regions. The results revealed that GFP-LuxR2 was enriched at the P1 and P2 regions of *luxR1* promoter (Fig. 7M), and GFP-LuxR1 was enriched at the P4 region of *ofaABC* promoter (Fig. 7P). As expected, deletion of the P1 and P2 regions of *luxR1* promoter, and the P4 region of *ofaABC* resulted in a significant decrease in GFP intensity (Fig. 7G and J). MST analysis also showed that deletion of these promoter regions abolished the corresponding bindings, thereby confirming this binding specificity (Fig. 7L and O). Collectively, these results establish a regulatory cascade in which LuxR2 activates *luxR1* transcription, and LuxR1 directly controls *ofa* operon expression, thereby driving orfamide A biosynthesis.

### The Gac–Rsm–LuxR cascade controls orfamide A-mediated biocontrol in the rhizosphere

**Figure 8.**
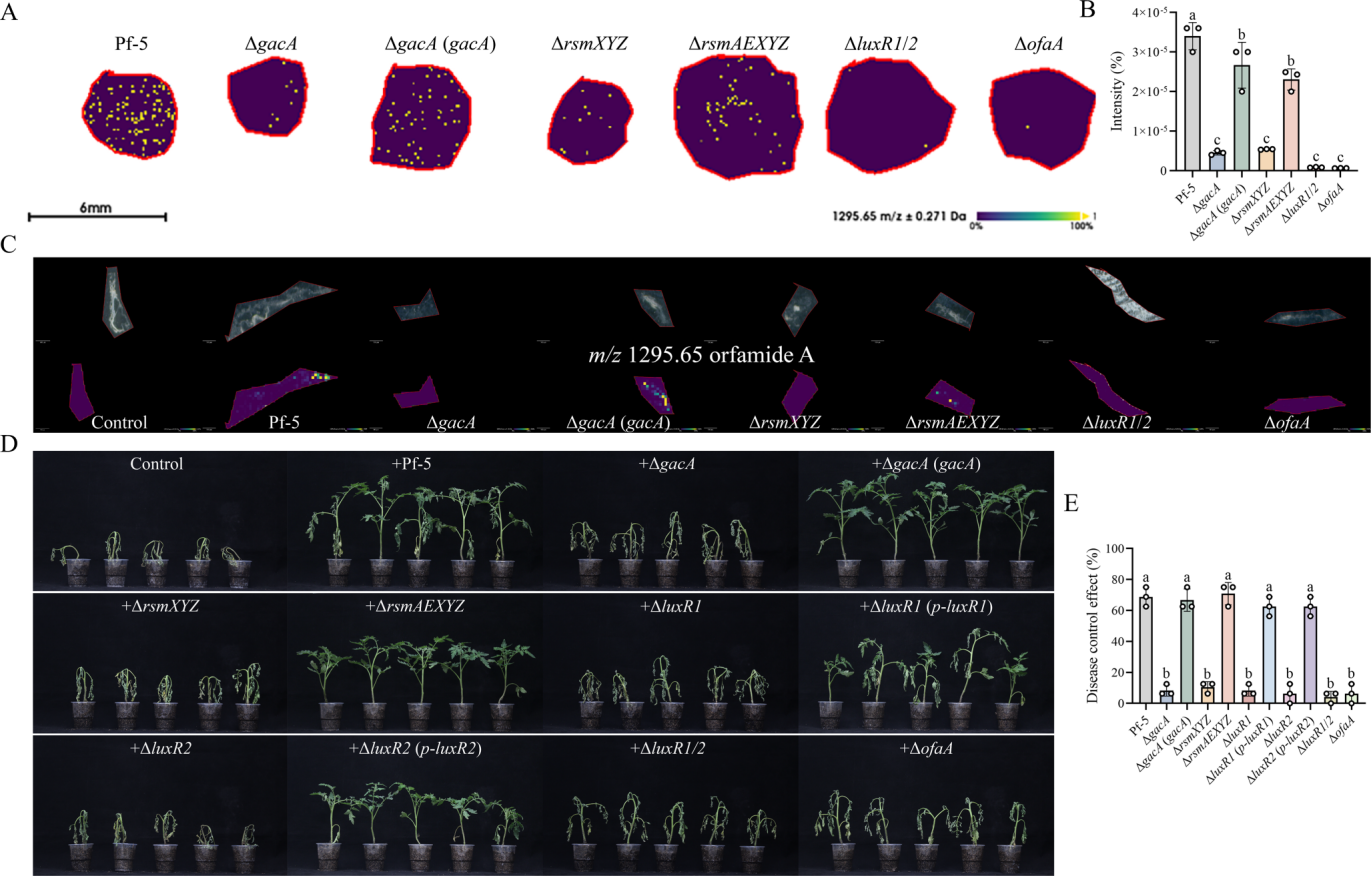
Orfamide A mediates the biocontrol activity of *P. protegens* Pf-5. (**A** and **B**) The orfamide A distribution of Pf-5, Δ*gacA*, Δ*gacA* (*gacA*), Δ*rsmXYZ*, Δ*rsmAEXYZ*, Δ*luxR1*/*2*, and Δ*ofaA* on MM medium; scale bar: 6 mm. MSI results were shown in panel (A), and the column diagram (B) showed the orfamide A production intensity. Data were presented as the mean ± SD from three independent experiments (*n* = 3) with similar results. Different lowercase letters within the same row indicate statistically significant differences. (**C**) Distribution of orfamide A in the tomato rhizosphere imaged by MALDI-MSI. Heat maps were applied to present the relative distributions and contents; scale bar: 500 μm. MSI shown in panels (A) and (C) were representative of three separate experiments with similar results. (**D** and **E**) Thirty-day-old tomato plants were infected with *R. solanacearum*, and relevant *Pseudomonas* strains. Disease control effect of the different treatments was exhibited in panel (D) and the control rate was described in panel (E). Control means only *R. solanacearum*; +Pf-5 means *R. solanacearum *+ Pf-5; +Δ*gacA* means *R. solanacearum*+Δ*gacA*; +Δ*gacA* (*gacA*) means *R. solanacearum*+Δ*gacA* (*gacA*); +Δ*rsmXYZ* means *R. solanacearum*+Δ*rsmXYZ*; +Δ*rsmAEXYZ* means *R. solanacearum*+Δ*rsmAEXYZ*; +Δ*luxR1* means *R. solanacearum*+Δ*luxR1*; +Δ*luxR1* (*p*-*luxR1*) means *R. solanacearum*+Δ*luxR1* (*p*-*luxR1*); +Δ*luxR2* means *R. solanacearum*+Δ*luxR2*; +Δ*luxR2* (*p*-*luxR2*) means *R. solanacearum*+Δ*luxR2* (*p*-*luxR2*); +Δ*luxR1*/*2* means *R. solanacearum*+Δ*luxR1*/*2*; +Δ*ofaA* means *R. solanacearum*+Δ*ofaA*. Disease assay shown in panel (D) were representative of three separate experiments with similar results. Data in (E) were presented as the mean ± SD from three independent experiments (*n* = 3) with similar results. Different lowercase letters within the same row indicate statistically significant differences.

Although the Gac–Rsm–LuxR regulatory cascade was genetically dissected under standard laboratory conditions, whether this regulation persists under ecologically relevant settings remains unclear. To address this, we assessed orfamide A production in a minimal medium (MM) designed to mimic the nutrient-limited environment of the rhizosphere. The MSI results showed that Pf-5, Δ*gacA* (*gacA*), and Δ*rsmAEXYZ* all produced readily detectable levels of orfamide A on MM medium, whereas the orfamide A production in Δ*gacA*, Δ*rsmXYZ*, Δ*luxR1/2*, and Δ*ofaA* strains was significantly diminished (Fig. 8A and B). To extend these findings to a more natural setting, we examined orfamide A production directly in the tomato rhizosphere using MSI. The production patterns observed in *planta* were consistent with those on MM and rich media (Fig. 8C and Supplementary Fig. S5).

To further determine whether the orfamide A production influence the biocontrol ability of relevant strains, greenhouse trials were performed using tomato seedlings challenged with the pathogen *Ralstonia solanacearum*. Compared to the control group inoculated with *R. solanacearum* alone, Pf-5, Δ*gacA* (*gacA*), Δ*rsmAEXYZ*, Δ*luxR1* (*p*-*luxR1*), and Δ*luxR2* (*p*-*luxR2*) achieved effective disease control rates of 71.9%, 68.8%, 68.8%, 59.4%, and 59.4%, respectively (Fig. 8D and E). In contrast, strains Δ*gacA*, Δ*rsmXYZ*, Δ*luxR1*, Δ*luxR2*, Δ*luxR1*/*2*, and Δ*ofaA*, which produce reduced levels of orfamide A due to mutations in the regulatory cascade, exhibited significantly diminished control rate of <10% (Fig. 8D and E).

Taken together, these results demonstrated that the Gac–Rsm–LuxR regulatory cascade modulates orfamide A production in the rhizosphere and, consequently, determines the biocontrol performance of Pf-5 against soilborne pathogens.

## Discussion

The Gac–Rsm cascade has garnered multiple attentions due to its universality and conservation across *Pseudomonas* spp., which plays a pivotal role in mediating the biosynthesis of secondary metabolites of PGPR, and virulence factors of phytopathogenic *Pseudomonas* spp., like *P. syringae* and *P. tolaasiii* [9, 21, 38]. Despite extensive researches on the Gac–Rsm cascade, a significant gap in our understanding of the molecular mechanisms regulating the biosynthesis of secondary metabolites still remains. Orfamide A is a biosurfactant produced by *Pseudomonas* spp. which related to the swarming motility and biocontrol function of *Pseudomonas* [24, 25]. In this study, we elucidate a complete Gac–Rsm–LuxR regulatory cascade that controls orfamide A biosynthesis in *P. protegens* Pf-5 and demonstrate its functionality in the rhizosphere environment. Our findings define a signalling pathway that transduces environmental cues into the production of a key biocontrol determinant.

It has been reported that small RNAs are involved in the regulation of diverse biological processes in *Pseudomonas* spp. [39]. For example, the sRNA PrrH and PA2952.1 of *P. aeruginosa* PAO1 inhibits the swimming and swarming motility of PAO1 [40]. Here, we found that GacS/A directly activate the transcription of three sRNAs (RsmX, RsmY, and RsmZ) in Pf-5 and, thereby influence its swarming motility and orfamide A production. sRNAs in the Rsm family are often described as functionally redundant due to their shared ability to sequester Rsm proteins [41]. Consistent with this, our study demonstrates that RsmY and RsmZ are functionally redundant in regulating orfamide A production. However, RsmX was found to play a compensatory role. Unlike RsmY and RsmZ, RsmX alone could not restore the Δ*rsmXYZ* phenotype. Its ability to complement only in Δ*rsmAYZ* and Δ*rsmEYZ* backgrounds suggests that RsmX is capable of titrating a single repressor RsmA or RsmE. Similar studies have reported that individual sRNAs can exert different degrees of function. For example, in *P. aeruginosa*, RsmY and RsmZ are the primary sRNAs that sequester repressor proteins RsmA/RsmF, and RsmV and RsmW appear to play smaller roles [42]. This partial redundancy probably reflects an adaptive strategy to maintain orfamide A production in the rhizosphere conditions. The possible reason of this phenomenon might be the different patterns of sRNAs expression levels. qRT-PCR results showed that among three sRNAs, expression levels of RsmX were lower than those of RsmY and RsmZ at 12 and 24 h (Supplementary Fig. S6). Our findings were supported by the report that the expression pattern of RsmV differs from those of RsmW, RsmY, and RsmZ, thereby each sRNAs play a distinct role in the Rsm system [42].

Analogous to CsrA in *E. coli*, which responds to the BarA/UvrY system, RsmA and RsmE in *Pseudomonas* regulate the most physiological processes, such as the extracellular enzyme AprA production and the motility [9, 10, 41, 43–45]. However, the targets of Rsm proteins modulating secondary metabolite biosynthesis have remained largely elusive. In the present study, we identify two transcription factors LuxR1 and LuxR2 as direct targets of RsmA and RsmE. To the best of our knowledge, this is the first report of Rsm proteins mediating LuxR-type transcriptional factor controlling secondary metabolite biosynthesis. Additionally, we prove that RsmA and RsmE exert repression function by binding to the *cis*-element GGA in the 5′UTR regions of LuxR1 and LuxR2 mRNA. It has been reported that RsmA and RsmE can modulate 2,4-DAPG production via bound of the transcribed mRNA of the *phlABCD* operon [37, 46]. However, in our case, RsmA and RsmE can’t directly bind to the *ofa* operon due to that the lack of *cis*-element GGA in its 5′UTR region. This divergence highlights the mechanistic flexibility of the Gac-Rsm system in controlling diverse secondary metabolites.

LuxR-type transcription factors are widely distributed in bacteria and play central roles in quorum sensing, virulence, and the control of secondary metabolite biosynthesis [47, 48]. It has been reported that deletion of *luxRup* and *luxRdown* in *Pseudomonas* CMR5c significantly reduced its motility and orfamide A production [24]. However, the specific regulation mechanism of two LuxR-type transcription factors remains unclear. Here, we revealed a two-tiered LuxR cascade in *P. protegens*: LuxR2 activates *luxR1* transcription, while LuxR1 directly bind the *ofaABC* promoter. This feedforward loop architecture sharpens the transcriptional response and prevents spurious activation, which may be essential for the orfamide A biosynthesis. Whether similar LuxR hierarchies are common in other exist in other *Pseudomonas* spp. gene clusters awaits further investigation.

Although the preceding results revealed the Gac–Rsm–LuxR regulatory cascade controlling orfamide A biosynthesis in *P. protegens* Pf-5, whether this signalling pathway is functional in the rhizosphere remained to be determined. Here, by using MM medium and tomato seedlings to mimic the nutrient-limited rhizosphere environment, we confirme that orfamide A production follows the same regulatory patterns observed under the laboratory conditions, indicating that the Gac–Rsm–LuxR regulatory cascade is functionally engaged in the rhizosphere. The results of greenhouse trials support this conclusion as well. Our findings establish a link between Gac/Rsm-mediated regulation and bacterial function in the rhizosphere, addressing the gap in understanding the ecological relevance of this signaling pathway.

In conclusion, we report a complete regulatory cascade Gac–Rsm–LuxR that can control orfamide A biosynthesis in *P. protegens* Pf-5 and demonstrate its functional importance in the rhizosphere. This hierarchical cascade research may be helpful for understanding how environmental signals are transduced through multiple regulatory layers to control specialized metabolite production in *Pseudomonads* and related bacteria.

## Supplementary Material

gkag501_Supplemental_Files

## Data Availability

The data underlying this article are available in the article and in its online supplementary material. Raw and processed data for RNA-seq are available in the GEO repository under the accession number GSE325590.
