# Supplementary material for: Regulation of cyclic lipopeptide orfamide A biosynthesis in Pseudomonas protegens by the Gac–Rsm–LuxR Cascade
Source: Nucleic Acids Res. 2026 May 20;54(10):gkag501. doi: 10.1093/nar/gkag501 (PMC13187841; doi:10.1093/nar/gkag501)
Supplement: gkag501_Supplemental_Files [file gkag501_supplemental_files.zip › Supplementary data.pdf]

Supplementary data

Regulation of Cyclic Lipopeptide Orfamide A Biosynthesis in *Pseudomonas protegens* by the Gac-Rsm-LuxR Cascade

AUTHORS

Ruoyi Wang[1,2,†], Chenjie Yu[1,2,4,†,*], Yan Song[1,2,†], Lulu Zhang[1,2], PingPing Li[1,2], Linli Zhu[1,2], Yali Fu[1,2], Rongshi Chen[1,2], Guohua Ding[6], Rainer Borriss[5], Xuewen Gao[1,2], Junqing Qiao[3,*], Qin Gu[1,2,*], Huijun Wu[1,2,*]

[1] The Sanya Institute of Nanjing Agricultural University, Nanjing Agricultural University, 572024, Sanya, China

[2] State Key Laboratory of Agricultural and Forestry Biosecurity, College of Plant Protection, Nanjing Agricultural University, 211800, Nanjing, China

[3] Institute of Plant Protection, Jiangsu Academy of Agricultural Sciences, 210014, Nanjing, China

[4] College of Horticulture Technology, Suzhou Polytechnic Institute of Agriculture, 215008, Suzhou, China

[5] Institute of Biology, Humboldt University, 10115, Berlin, Germany

[6] Modern Agricultural Analysis and Testing Center, Nanjing Agricultural University, 211800, Nanjing, China

* To whom correspondence should be addressed. Tel: +86 13770914062; Email: hjwu@njau.edu.cn

Correspondence may also be addressed to Qin Gu. Email: guqin@njau.edu.cn

Correspondence may also be addressed to Junqing Qiao. Email: qiaojunqing@jaas.ac.cn

Correspondence may also be addressed to Chenjie Yu. Email: yuchenjie0501@163.com

† The authors wish it to be known that, in their opinion, the first three authors should be regarded as Joint First Authors.

Table S1 Strains and plasmids used in this study

Strains and Plasmids	Genotype and relevant characteristics	Source
Pseudomonas protegens		
Pf-5	Wild-type; Amp^r	Laboratory stock
Δ*gacA*	Pf-5 derivative strain containing an in-frame deletion of *gacA* in the chromosome; Amp^r	This study
Δ*gacA* (*gacA*)	Δ*gacA* derivative strain containing pBBR1MCS-5-*p-gacA* vector; Amp^r, Gm^r	This study
Pf-5 (pBBR, pME6522)	Pf-5 derivative strain containing pBBR1MCS-5 and pME6522 vector used for luciferase activity detection; Amp^r, Gm^r, Tc^r	This study
Pf-5 (pBBR, pME6522-*prsmX-lux*)	Pf-5 derivative strain containing pBBR1MCS-5 and pME6522-*prsmX-lux* used for luciferase activity detection; Amp^r, Gm^r, Tc^r	This study
Δ*gacA* (pBBR, pME6522-*prsmX-lux*)	Δ*gacA* derivative strain containing pBBR1MCS-5 and pME6522-*prsmX-lux* used for luciferase activity detection; Amp^r, Gm^r, Tc^r	This study
Δ*gacA* (pBBR-*p-gacA*, pME6522-*prsmX-lux*)	Δ*gacA* (*gacA*) derivative strain containing pME6522-*prsmX-lux* used for luciferase activity detection; Amp^r, Gm^r, Tc^r	This study
Pf-5 (pBBR, pME6522-*prsmY-lux*)	Pf-5 derivative strain containing pBBR1MCS-5 and pME6522-*prsmY-lux* used for luciferase activity detection; Amp^r, Gm^r, Tc^r	This study
Δ*gacA* (pBBR, pME6522-*prsmY-lux*)	Δ*gacA* derivative strain containing pBBR1MCS-5 and pME6522-*prsmY-lux* used for luciferase activity detection; Amp^r, Gm^r, Tc^r	This study
Δ*gacA* (pBBR-*p-gacA*, pME6522-*prsmY-lux*)	Δ*gacA* (*gacA*) derivative strain containing pME6522-*prsmY-lux* used for luciferase activity detection; Amp^r, Gm^r, Tc^r	This study
Pf-5 (pBBR, pME6522-*prsmZ-lux*)	Pf-5 derivative strain containing pBBR1MCS-5 and pME6522-*prsmZ-lux* used for luciferase activity detection; Amp^r, Gm^r, Tc^r	This study
Δ*gacA* (pBBR, pME6522-*prsmZ-lux*)	Δ*gacA* derivative strain containing pBBR1MCS-5 and pME6522-*prsmZ-lux* used for luciferase activity detection; Amp^r, Gm^r, Tc^r	This study
Δ*gacA* (pBBR-*p-gacA*, pME6522-*prsmZ-lux*)	Δ*gacA* (*gacA*) derivative strain containing pME6522-*prsmZ-lux* used for luciferase activity detection; Amp^r, Gm^r, Tc^r	This study
Δ*rsmX*	Pf-5 derivative strain containing an in-frame deletion of *rsmX* in the chromosome; Amp^r	This study
Δ*rsmY*	Pf-5 derivative strain containing an in-frame deletion of *rsmY* in the chromosome; Amp^r	This study
Δ*rsmZ*	Pf-5 derivative strain containing an in-frame deletion of *rsmZ* in the chromosome; Amp^r	This study
Δ*rsmXY*	Pf-5 derivative strain containing an in-frame deletion of *rsmX* and *rsmY* in the chromosome; Amp^r	This study
Δ*rsmXZ*	Pf-5 derivative strain containing an in-frame deletion of *rsmX* and *rsmZ* in the chromosome; Amp^r	This study
Δ*rsmYZ*	Pf-5 derivative strain containing an in-frame deletion of *rsmY* and *rsmZ* in the chromosome; Amp^r	This study
Δ*rsmXYZ*	Pf-5 derivative strain containing an in-frame deletion of *rsmX*,	This study

	rsmY and *rsmZ* in the chromosome; Amp^r^	
Δ*rsmAYZ*	Pf-5 derivative strain containing an in-frame deletion of *rsmA*, *rsmY* and *rsmZ* in the chromosome; Amp^r^	This study
Δ*rsmEYZ*	Pf-5 derivative strain containing an in-frame deletion of *rsmE*, *rsmY* and *rsmZ* in the chromosome; Amp^r^	This study
Δ*rsmAEYZ*	Pf-5 derivative strain containing an in-frame deletion of *rsmA*, *rsmE*, *rsmY* and *rsmZ* in the chromosome; Amp^r^	This study
Δ*rsmAXYZ*	Pf-5 derivative strain containing an in-frame deletion of *rsmA*, *rsmX*, *rsmY* and *rsmZ* in the chromosome; Amp^r^	This study
Δ*rsmEXYZ*	Pf-5 derivative strain containing an in-frame deletion of *rsmE*, *rsmX*, *rsmY* and *rsmZ* in the chromosome; Amp^r^	This study
Δ*rsmAEXYZ*	Pf-5 derivative strain containing an in-frame deletion of *rsmA*, *rsmE*, *rsmX*, *rsmY* and *rsmZ* in the chromosome; Amp^r^	This study
Δ*rsmAE*	Pf-5 derivative strain containing an in-frame deletion of *rsmA* and *rsmE* in the chromosome; Amp^r^	This study
Pf-5 (pBBR)	Pf-5 derivative strain containing pBBR1MCS-5 vector used for swarming motility assays; Amp^r^, Gm^r^	This study
Δ*rsmXYZ* (pBBR)	Δ*rsmXYZ* derivative strain containing pBBR1MCS-5 used for swarming motility assays; Amp^r^, Gm^r^	This study
Δ*rsmXYZ* (*rsmX*)	Δ*rsmXYZ* derivative strain containing pBBR1MCS-5-*p-rsmX* used for swarming motility assays; Amp^r^, Gm^r^	This study
Δ*rsmXYZ* (*rsmY*)	Δ*rsmXYZ* derivative strain containing pBBR1MCS-5-*p-rsmY* used for swarming motility assays; Amp^r^, Gm^r^	This study
Δ*rsmXYZ* (*rsmZ*)	Δ*rsmXYZ* derivative strain containing pBBR1MCS-5-*p-rsmZ* used for swarming motility assays; Amp^r^, Gm^r^	This study
Δ*rsmXYZ* (*rsmY*$^{\Delta GGA}$)	Δ*rsmXYZ* derivative strain containing pBBR1MCS-5-*p-rsmY*$^{\Delta GGA}$ used for swarming motility assays; Amp^r^, Gm^r^	This study
Δ*rsmXYZ* (*rsmZ*$^{\Delta GGA}$)	Δ*rsmXYZ* derivative strain containing pBBR1MCS-5-*p-rsmZ*$^{\Delta GGA}$ used for swarming motility assays; Amp^r^, Gm^r^	This study
Pf-5 (pME6015-*lux*)	Pf-5 derivative strain containing pME6015-*lux* used for luciferase activity detection; Amp^r^, Tc^r^	This study
Pf-5 (pME6015-*pluxR1-lux*)	Pf-5 derivative strain containing pME6015-*pluxR1-lux* used for luciferase activity detection; Amp^r^, Tc^r^	This study
Δ*rsmYZ* (pME6015-*pluxR1-lux*)	Δ*rsmYZ* derivative strain containing pME6015-*pluxR1-lux* used for luciferase activity detection; Amp^r^, Tc^r^	This study
Δ*rsmAYZ* (pME6015-*pluxR1-lux*)	Δ*rsmAYZ* derivative strain containing pME6015-*pluxR1-lux* used for luciferase activity detection; Amp^r^, Tc^r^	This study
Δ*rsmEYZ* (pME6015-*pluxR1-lux*)	Δ*rsmEYZ* derivative strain containing pME6015-*pluxR1-lux* used for luciferase activity detection; Amp^r^, Tc^r^	This study
Δ*rsmAEYZ* (pME6015-*pluxR1-lux*)	Δ*rsmAEYZ* derivative strain containing pME6015-*pluxR1-lux* used for luciferase activity detection; Amp^r^, Tc^r^	This study
Δ*rsmXYZ* (pME6015-*pluxR1-lux*)	Δ*rsmXYZ* derivative strain containing pME6015-*pluxR1-lux* used for luciferase activity detection; Amp^r^, Tc^r^	This study
Δ*rsmAEXYZ* (pME6015-	Δ*rsmAEXY* derivative strain containing pME6015-*pluxR1-lux*	This study

pluxR1-lux)	used for luciferase activity detection; Amp^r, Tc^r	
Δ*rsmAE* (pME6015-*pluxR1-lux*)	Δ*rsmAE* derivative strain containing pME6015-*pluxR1-lux* used for luciferase activity detection; Amp^r, Tc^r	This study
Pf-5 (pME6015-*pluxR2-lux*)	Pf-5 derivative strain containing pME6015-*pluxR2-lux* used for luciferase activity detection; Amp^r, Tc^r	This study
Δ*rsmYZ* (pME6015-*pluxR2-lux*)	Δ*rsmYZ* derivative strain containing pME6015-*pluxR2-lux* used for luciferase activity detection; Amp^r, Tc^r	This study
Δ*rsmAYZ* (pME6015-*pluxR2-lux*)	Δ*rsmAYZ* derivative strain containing pME6015-*pluxR2-lux* used for luciferase activity detection; Amp^r, Tc^r	This study
Δ*rsmEYZ* (pME6015-*pluxR2-lux*)	Δ*rsmEYZ* derivative strain containing pME6015-*pluxR2-lux* used for luciferase activity detection; Amp^r, Tc^r	This study
Δ*rsmAEYZ* (pME6015-*pluxR2-lux*)	Δ*rsmAEYZ* derivative strain containing pME6015-*pluxR2-lux* used for luciferase activity detection; Amp^r, Tc^r	This study
Δ*rsmXYZ* (pME6015-*pluxR2-lux*)	Δ*rsmXYZ* derivative strain containing pME6015-*pluxR2-lux* used for luciferase activity detection; Amp^r, Tc^r	This study
Δ*rsmAEXYZ* (pME6015-*pluxR2-lux*)	Δ*rsmAEXY* derivative strain containing pME6015-*pluxR2-lux* used for luciferase activity detection; Amp^r, Tc^r	This study
Δ*rsmAE* (pME6015-*pluxR2-lux*)	Δ*rsmAE* derivative strain containing pME6015-*pluxR2-lux* used for luciferase activity detection; Amp^r, Tc^r	This study
Pf-5 (pME6015-*pofaABC-lux*)	Pf-5 derivative strain containing pME6015-*pofaABC-lux* used for luciferase activity detection; Amp^r, Tc^r	This study
Δ*rsmYZ* (pME6015-*pofaABC-lux*)	Δ*rsmYZ* derivative strain containing pME6015-*pofaABC-lux* used for luciferase activity detection; Amp^r, Tc^r	This study
Δ*rsmAYZ* (pME6015-*pofaABC-lux*)	Δ*rsmAYZ* derivative strain containing pME6015-*pofaABC-lux* used for luciferase activity detection; Amp^r, Tc^r	This study
Δ*rsmEYZ* (pME6015-*pofaABC-lux*)	Δ*rsmEYZ* derivative strain containing pME6015-*pofaABC-lux* used for luciferase activity detection; Amp^r, Tc^r	This study
Δ*rsmAEYZ* (pME6015-*pofaABC-lux*)	Δ*rsmAEYZ* derivative strain containing pME6015-*pofaABC-lux* used for luciferase activity detection; Amp^r, Tc^r	This study
Δ*rsmXYZ* (pME6015-*pofaABC-lux*)	Δ*rsmXYZ* derivative strain containing pME6015-*pofaABC-lux* used for luciferase activity detection; Amp^r, Tc^r	This study
Δ*rsmAEXYZ* (pME6015-*pofaABC-lux*)	Δ*rsmAEXY* derivative strain containing pME6015-*pofaABC-lux* used for luciferase activity detection; Amp^r, Tc^r	This study
Δ*rsmAE* (pME6015-*pofaABC-lux*)	Δ*rsmAE* derivative strain containing pME6015-*pofaABC-lux* used for luciferase activity detection; Amp^r, Tc^r	This study
Pf-5 (pBBR, pME6015-*pluxR1*-GFP)	Pf-5 derivative strain containing pBBR1MCS-5 and pME6015-*pluxR1*-GFP used for GFP intensity detection; Amp^r, Gm^r, Tc^r	This study
Δ*gacA* (pBBR, pME6015-*pluxR1*-GFP)	Δ*gacA* derivative strain containing pBBR1MCS-5 and pME6015-*pluxR1*-GFP used for GFP intensity detection; Amp^r, Gm^r, Tc^r	This study
Δ*gacA* (pBBR-*p-gacA*, pME6015-*pluxR1*-GFP)	Δ*gacA* (*gacA*) derivative strain containing pME6015-*pluxR1*-GFP used for GFP intensity detection; Amp^r, Gm^r, Tc^r	This study
Δ*rsmXYZ* (pBBR, pME6015-*pluxR1*-GFP)	Δ*rsmXYZ* derivative strain containing pBBR1MCS-5 and pME6015-*pluxR1*-GFP used for GFP intensity detection; Amp^r,	This study

	Gm^r, Tc^r	
Δ*rsmAEXYZ* (pBBR, pME6015-*pluxR1*-GFP)	Δ*rsmAEXYZ* derivative strain containing pBBR1MCS-5 and pME6015-*pluxR1*-GFP used for GFP intensity detection; Amp^r, Gm^r, Tc^r	This study
Pf-5 (pBBR, pME6015-*pluxR1**-GFP)	Pf-5 derivative strain containing pBBR1MCS-5 and pME6015-*pluxR1**-GFP used for GFP intensity detection; Amp^r, Gm^r, Tc^r	This study
Δ*gacA* (pBBR, pME6015-*pluxR1**-GFP)	Δ*gacA* derivative strain containing pBBR1MCS-5 and pME6015-*pluxR1**-GFP used for GFP intensity detection; Amp^r, Gm^r, Tc^r	This study
Δ*gacA* (pBBR-*p-gacA*, pME6015-*pluxR1**-GFP)	Δ*gacA* (*gacA*) derivative strain containing pME6015-*pluxR1**-GFP used for GFP intensity detection; Amp^r, Gm^r, Tc^r	This study
Δ*rsmXYZ* (pBBR, pME6015-*pluxR1**-GFP)	Δ*rsmXYZ* derivative strain containing pBBR1MCS-5 and pME6015-*pluxR1**-GFP used for GFP intensity detection; Amp^r, Gm^r, Tc^r	This study
Δ*rsmAEXYZ* (pBBR, pME6015-*pluxR1**-GFP)	Δ*rsmAEXYZ* derivative strain containing pBBR1MCS-5 and pME6015-*pluxR1**-GFP used for GFP intensity detection; Amp^r, Gm^r, Tc^r	This study
Pf-5 (pBBR, pME6015-*pluxR2*-GFP)	Pf-5 derivative strain containing pBBR1MCS-5 and pME6015-*pluxR2*-GFP used for GFP intensity detection; Amp^r, Gm^r, Tc^r	This study
Δ*gacA* (pBBR, pME6015-*pluxR2*-GFP)	Δ*gacA* derivative strain containing pBBR1MCS-5 and pME6015-*pluxR2*-GFP used for GFP intensity detection; Amp^r, Gm^r, Tc^r	This study
Δ*gacA* (pBBR-*p-gacA*, pME6015-*pluxR2*-GFP)	Δ*gacA* (*gacA*) derivative strain containing pME6015-*pluxR2*-GFP used for GFP intensity detection; Amp^r, Gm^r, Tc^r	This study
Δ*rsmXYZ* (pBBR, pME6015-*pluxR2*-GFP)	Δ*rsmXYZ* derivative strain containing pBBR1MCS-5 and pME6015-*pluxR2*-GFP used for GFP intensity detection; Amp^r, Gm^r, Tc^r	This study
Δ*rsmAEXYZ* (pBBR, pME6015-*pluxR2*-GFP)	Δ*rsmAEXYZ* derivative strain containing pBBR1MCS-5 and pME6015-*pluxR2*-GFP used for GFP intensity detection; Amp^r, Gm^r, Tc^r	This study
Pf-5 (pBBR, pME6015-*pluxR2**-GFP)	Pf-5 derivative strain containing pBBR1MCS-5 and pME6015-*pluxR2**-GFP used for GFP intensity detection; Amp^r, Gm^r, Tc^r	This study
Δ*gacA* (pBBR, pME6015-*pluxR2**-GFP)	Δ*gacA* derivative strain containing pBBR1MCS-5 and pME6015-*pluxR2**-GFP used for GFP intensity detection; Amp^r, Gm^r, Tc^r	This study
Δ*gacA* (pBBR-*p-gacA*, pME6015-*pluxR2**-GFP)	Δ*gacA* (*gacA*) derivative strain containing pME6015-*pluxR2**-GFP used for GFP intensity detection; Amp^r, Gm^r, Tc^r	This study
Δ*rsmXYZ* (pBBR, pME6015-*pluxR2**-GFP)	Δ*rsmXYZ* derivative strain containing pBBR1MCS-5 and pME6015-*pluxR2**-GFP used for GFP intensity detection; Amp^r, Gm^r, Tc^r	This study
Δ*rsmAEXYZ* (pBBR, pME6015-*pluxR2**-GFP)	Δ*rsmAEXYZ* derivative strain containing pBBR1MCS-5 and pME6015-*pluxR2**-GFP used for GFP intensity detection; Amp^r, Gm^r, Tc^r	This study
Pf-5 (pME6032)	Pf-5 derivative strain containing pME6032 vector used for	This study

	orfamide A production detection; Amp^r, Tc^r	
Δ*luxR1* (pME6032)	Δ*luxR1* derivative strain containing pME6032 vector used for orfamide A production detection; Amp^r, Tc^r	This study
Δ*luxR2* (pME6032)	Δ*luxR2* derivative strain containing pME6032 vector used for orfamide A production detection; Amp^r, Tc^r	This study
Δ*luxR1* (*p*IPTG-*luxR1*)	Δ*luxR1* derivative strain containing pME6032-*luxR1* vector used for orfamide A production detection; Amp^r, Tc^r	This study
Δ*luxR2* (*p*IPTG-*luxR2*)	Δ*luxR2* derivative strain containing pME6032-*luxR2* vector used for orfamide A production detection; Amp^r, Tc^r	This study
Δ*luxR1/2*	Pf-5 derivative strain containing an in-frame deletion of *luxR1* and *luxR2* in the chromosome; Amp^r	This study
Δ*luxR1/2* (pBBR, pME6032)	Δ*luxR1/2* derivative strain containing pBBR1MCS-5 and pME6032 vector used for orfamide A production detection; Amp^r, Tc^r, Gm^r	This study
Δ*luxR1/2* (*p*IPTG-*luxR1*)	Δ*luxR1/2* derivative strain containing pME6032-*luxR1* vector used for orfamide A production detection; Amp^r, Tc^r	This study
Δ*luxR1/2* (*p*IPTG-*luxR2*)	Δ*luxR1/2* derivative strain containing pME6032-*luxR2* vector used for orfamide A production detection; Amp^r, Tc^r	This study
Δ*luxR1/2* (*p*-*luxR1*)	Δ*luxR1/2* derivative strain containing pBBR1MCS-5-*p*-*luxR1* vector used for orfamide A production detection; Amp^r, Gm^r	This study
Δ*luxR1/2* (*p*IPTG-*luxR2*, *np*-*luxR1*)	Δ*luxR1/2* derivative strain containing pME6032-*luxR2* and pBBR1MCS-5-*luxR1* vector used for orfamide A production detection; Amp^r, Tc^r, Gm^r	This study
Δ*luxR1/2* (*p*IPTG-*luxR2*, *p*-*luxR1*)	Δ*luxR1/2* derivative strain containing pME6032-*luxR2* and pBBR1MCS-5-*p*-*luxR1* vector used for orfamide A production detection; Amp^r, Tc^r, Gm^r	This study
Pf-5 (pBBR, pME6015-*pluxR2-lacZ*)	Pf-5 derivative strain containing pBBR1MCS-5 and pME6015-*pluxR2-lacZ* used for β-galactosidase activity detection; Amp^r, Tc^r, Gm^r	This study
Δ*luxR1* (pBBR, pME6015-*pluxR2-lacZ*)	Δ*luxR1* derivative strain containing pBBR1MCS-5 and pME6015-*pluxR2-lacZ* used for β-galactosidase activity detection; Amp^r, Tc^r, Gm^r	This study
Δ*luxR1* (pBBR-*luxR1*, pME6015-*pluxR2-lacZ*)	Δ*luxR1* derivative strain containing pBBR1MCS-5-*luxR1* and pME6015-*pluxR2-lacZ* used for β-galactosidase activity detection; Amp^r, Tc^r, Gm^r	This study
Pf-5 (pBBR, pME6015-*pluxR1-lacZ*)	Pf-5 derivative strain containing pBBR1MCS-5 and pME6015-*pluxR1-lacZ* used for β-galactosidase activity detection; Amp^r, Tc^r, Gm^r	This study
Δ*luxR2* (pBBR, pME6015-*pluxR1-lacZ*)	Δ*luxR2* derivative strain containing pBBR1MCS-5 and pME6015-*pluxR1-lacZ* used for β-galactosidase activity detection; Amp^r, Tc^r, Gm^r	This study
Δ*luxR2* (pBBR-*p*-*luxR2*, pME6015-*pluxR1-lacZ*)	Δ*luxR2* derivative strain containing pBBR1MCS-5-*p*-*luxR2* and pME6015-*pluxR1-lacZ* used for β-galactosidase activity detection; Amp^r, Tc^r, Gm^r	This study

Pf-5 (pBBR, pME6015-*pofaABC-lacZ*)	Pf-5 derivative strain containing pBBR1MCS-5 and pME6015-*pofaABC-lacZ* used for β-galactosidase activity detection; Amp^r, Tc^r, Gm^r	This study
Δ*luxR1* (pBBR, pME6015-*pofaABC-lacZ*)	Δ*luxR1* derivative strain containing pBBR1MCS-5 and pME6015-*pofaABC-lacZ* used for β-galactosidase activity detection; Amp^r, Tc^r, Gm^r	This study
Δ*luxR1* (pBBR-*p-luxR1*, pME6015-*pofaABC-lacZ*)	Δ*luxR1* derivative strain containing pBBR1MCS-5-*p-luxR1* and pME6015-*pofaABC-lacZ* used for β-galactosidase activity detection; Amp^r, Tc^r, Gm^r	This study
Δ*luxR2* (pBBR, pME6015-*pluxR1*-GFP)	Δ*luxR2* derivative strain containing pBBR1MCS-5 and pME6015-*pluxR1*-GFP used for GFP intensity detection; Amp^r, Gm^r, Tc^r	This study
Δ*luxR2* (pBBR-*p-luxR2*, pME6015-*pluxR1*-GFP)	Δ*luxR2* derivative strain containing pBBR1MCS-5-*p-luxR2* and pME6015-*pluxR1*-GFP used for GFP intensity detection; Amp^r, Gm^r, Tc^r	This study
Pf-5 (pBBR, pME6015-*pluxR1*$^{\Delta P1\text{-}2}$-GFP)	Pf-5 derivative strain containing pBBR1MCS-5 and pME6015-*pluxR1*$^{\Delta P1\text{-}2}$-GFP used for GFP intensity detection; Amp^r, Gm^r, Tc^r	This study
Δ*luxR2* (pBBR, pME6015-*pluxR1*$^{\Delta P1\text{-}2}$-GFP)	Δ*luxR2* derivative strain containing pBBR1MCS-5 and pME6015-*pluxR1*$^{\Delta P1\text{-}2}$-GFP used for GFP intensity detection; Amp^r, Gm^r, Tc^r	This study
Δ*luxR2* (pBBR-*p-luxR2*, pME6015-*pluxR1*$^{\Delta P1\text{-}2}$-GFP)	Δ*luxR2* derivative strain containing pBBR1MCS-5-*p-luxR2* and pME6015-*pluxR1*$^{\Delta P1\text{-}2}$-GFP used for GFP intensity detection; Amp^r, Gm^r, Tc^r	This study
Pf-5 (pBBR, pME6015-*pofaABC*-GFP)	Pf-5 derivative strain containing pBBR1MCS-5 and pME6015-*pofaABC*-GFP used for GFP intensity detection; Amp^r, Gm^r, Tc^r	This study
Δ*luxR1* (pBBR, pME6015-*pofaABC*-GFP)	Δ*luxR1* derivative strain containing pBBR1MCS-5 and pME6015-*pofaABC*-GFP used for GFP intensity detection; Amp^r, Gm^r, Tc^r	This study
Δ*luxR1* (pBBR-*p-luxR1*, pME6015-*pofaABC*-GFP)	Δ*luxR1* derivative strain containing pBBR1MCS-5-*p-luxR1* and pME6015-*pofaABC*-GFP used for GFP intensity detection; Amp^r, Gm^r, Tc^r	This study
Pf-5 (pBBR, pME6015-*pofaABC*$^{\Delta P4}$-GFP)	Pf-5 derivative strain containing pBBR1MCS-5 and pME6015-*pofaABC*$^{\Delta P4}$-GFP used for GFP intensity detection; Amp^r, Gm^r, Tc^r	This study
Δ*luxR1* (pBBR, pME6015-*pofaABC*$^{\Delta P4}$-GFP)	Δ*luxR1* derivative strain containing pBBR1MCS-5 and pME6015-*pofaABC*$^{\Delta P4}$-GFP used for GFP intensity detection; Amp^r, Gm^r, Tc^r	This study
Δ*luxR1* (pBBR-*p-luxR1*, pME6015-*pofaABC*$^{\Delta P4}$-GFP)	Δ*luxR1* derivative strain containing pBBR1MCS-5-*p-luxR1* and pME6015-*pofaABC*$^{\Delta P4}$-GFP used for GFP intensity detection; Amp^r, Gm^r, Tc^r	This study
Pf-5 (pME6015-*luxR1*-GFP)	Pf-5 derivative strain containing pME6015-*luxR1*-GFP used for ChIP-qPCR assays; Amp^r, Tc^r	This study
Pf-5 (pME6015-*luxR2*-GFP)	Pf-5 derivative strain containing pME6015-*luxR2*-GFP used for	This study

	ChIP-qPCR assays; Amp^r, Tc^r	
Escherichia coli		
Top10	Host strain for molecular cloning	TaKaRa Company
Bl21 (DE3)	Host strain for protein expression	TaKaRa Company
Plasmids		
pK18mobsacB	Suicide vector used for gene knockout; Km^r	Laboratory stock
pRK2013	Conjugation plasmid used for gene knockout; Km^r	Laboratory stock
pBBR1MCS-5	Broad-host-range cloning vector used for gene complement; Gm^r	Laboratory stock
pME6032	pVS1-p15A *E. coli-pseudomonas* shuttle vector, *lacI*^{Q}-P$_{tac}$ expression vector, used for gene complement; Tc^r	Laboratory stock
pME6522-*lux*	pVS1-p15A *E. coli-pseudomonas* shuttle vector for translational *lux* fusions and promoter probing, used for luciferase activity detection; Tc^r	Laboratory stock
pME6015-*lux*	pVS1-p15A *E. coli-pseudomonas* shuttle vector for translational *lux* fusions and promoter probing, used for luciferase activity detection; Tc^r	Laboratory stock
pME6015-GFP	pVS1-p15A *E. coli-pseudomonas* shuttle vector for translational *lux* fusions and promoter probing, used for GFP activity detection; Tc^r	Laboratory stock
pME6015-*lacZ*	pVS1-p15A *E. coli-pseudomonas* shuttle vector for translational *lacZ* fusions and promoter probing, used for β-galactosidase activity detection; Tc^r	Laboratory stock
pET28a	Vector used for protein expression; Km^r	Laboratory stock
pOPTHis	Vector used for protein expression; Amp^r	Laboratory stock
pK18-*gacA*	pK18mobsacB containing overlapping fragments of upstream and downstream of *gacA*; Km^r	This study
pK18-*rsmX*	pK18mobsacB containing overlapping fragments of upstream and downstream of *rsmX*; Km^r	This study
pK18-*rsmY*	pK18mobsacB containing overlapping fragments of upstream and downstream of *rsmY*; Km^r	This study
pK18-*rsmZ*	pK18mobsacB containing overlapping fragments of upstream and downstream of *rsmZ*; Km^r	This study
pK18-*rsmA*	pK18mobsacB containing overlapping fragments of upstream and downstream of *rsmA*; Km^r	This study
pK18-*rsmE*	pK18mobsacB containing overlapping fragments of upstream and downstream of *rsmE*; Km^r	This study
pK18-*luxR1*	pK18mobsacB containing overlapping fragments of upstream and downstream of *luxR1*; Km^r	This study
pK18-*luxR2*	pK18mobsacB containing overlapping fragments of upstream and downstream of *luxR2*; Km^r	This study
pBBR-*p-gacA*	pBBR1MCS-5 containing *gacA* with its native promoter; Gm^r	This study
pME6522-*prsmX-lux*	pME6522 containing the promoter of *rsmX* fused with *lux*; Tc^r	This study

pME6522-*prsmY-lux*	pME6522 containing the promoter of *rsmY* fused with *lux*; Tc^r	This study
pME6522-*prsmZ-lux*	pME6522 containing the promoter of *rsmZ* fused with *lux*; Tc^r	This study
pME6015-*pluxR1-lux*	pME6015 containing the promoter of *luxR1* fused with *lux*; Tc^r	This study
pME6015-*pluxR2-lux*	pME6015 containing the promoter of *luxR2* fused with *lux*; Tc^r	This study
pME6015-*pofaABC-lux*	pME6015 containing the promoter of *ofaABC* fused with *lux*; Tc^r	This study
pME6015-*pluxR1*-GFP	pME6015 containing the promoter of *luxR1* fused with GFP; Tc^r	This study
pME6015-*pluxR2*-GFP	pME6015 containing the promoter of *luxR2* fused with GFP; Tc^r	This study
pME6015-*pofaABC*-GFP	pME6015 containing the promoter of *ofaABC* fused with GFP; Tc^r	This study
pME6015-*pluxR1**-GFP	pME6015 containing the promoter of *luxR1* with a substitution of GGA to AGA fused with GFP; Tc^r	This study
pME6015-*pluxR2**-GFP	pME6015 containing the promoter of *luxR2* with a substitution of GGA to CCA fused with GFP; Tc^r	This study
pME6015-*pluxR1*$^{\Delta P1-2}$-GFP	pME6015 containing the promoter of *luxR1* without the P1 and P2 region fused with GFP; Tc^r	This study
pME6015-*pofaABC*$^{\Delta P4}$-GFP	pME6015 containing the promoter of *ofaABC* without the P4 region fused with GFP; Tc^r	This study
pME6015-*luxR1*-GFP	pME6015 containing the *luxR1* gene fused with GFP; Tc^r	This study
pME6015-*luxR2*-GFP	pME6015 containing the *luxR2* gene fused with GFP; Tc^r	This study
pET28a-*rsmA*	pET28a containing *rsmA*, used for the expression of RsmA protein; Km^r	This study
pET28a-*rsmE*	pET28a containing *rsmE*, used for the expression of RsmE protein; Km^r	This study
pBBR-*p-rsmX*	pBBR1MCS-5 containing *rsmX* with its native promoter; Gm^r	This study
pBBR-*p-rsmY*	pBBR1MCS-5 containing *rsmY* with its native promoter; Gm^r	This study
pBBR-*p-rsmZ*	pBBR1MCS-5 containing *rsmZ* with its native promoter; Gm^r	This study
pBBR-*p-rsmY*$^{\Delta GGA}$	pBBR1MCS-5 harbouring *rsmY* containing its native promoter and without the Rsm binding motif; Gm^r	This study
pBBR-*p-rsmZ*$^{\Delta GGA}$	pBBR1MCS-5 harbouring *rsmZ* containing its native promoter and without the Rsm binding motif; Gm^r	This study
pBBR-*p-luxR1*	pBBR1MCS-5 containing *luxR1* with its native promoter; Gm^r	This study
pBBR-*p-luxR2*	pBBR1MCS-5 containing *luxR2* with its native promoter; Gm^r	This study
pBBR-*luxR1*	pBBR1MCS-5 containing *luxR1*; Gm^r	This study
pBBR-*luxR2*	pBBR1MCS-5 containing *luxR2*; Gm^r	This study
pME6032-*luxR1*	pME6032 containing *luxR1*; Tc^r	This study
pME6032-*luxR2*	pME6032 containing *luxR2*; Tc^r	This study
pME6015-*pluxR1-lacZ*	pME6015 containing the promoter of *luxR1* fused with *lacZ*; Tc^r	This study
pME6015-*pofaABC-lacZ*	pME6015 containing the promoter of *ofaABC* fused with *lacZ*; Tc^r	This study
pOPTHis-*luxR1*	pOPTHis containing the *luxR1* gene, used for the expression of LuxR1 protein; Amp^r	This study

pOPTHis-*luxR2*	pOPTHis containing the *luxR2* gene, used for the expression of LuxR2 protein; Amp^r	This study

Resistance marker: Amp^r, ampicillin resistance; Km^r, kanamycin resistance; Gm^r, gentamicin resistance; Tc^r, tetracycline resistance.

Table S2 Primers used in this study

Primers	Sequence
Construct mutants	
gacA up F	ACGCGTCGACCGGGCATAGTTCAAAACC (*Sal*I)
gacA up R	TGACGAACCGCCAATAGCGCAGACACCTCGCGATAT
gacA down F	ATATCGCGAGGTGTCTGCGCTATTGGCGGTTCGTCA
gacA down R	CCCAAGCTTCGTAGGGGTAGGACTTATC (*Hin*dIII)
gacA out F	TTTGCCTTTCTTGCGGCCT
gacA out R	TTCTGCAACAGGCTGAGG
rsmX up F	ACGCGTCGACACGATGAGGTCAGCCATA (*Sal*I)
rsmX up R	CAGGCACGAAATGCCGGAGATTAAACACAAAGCCCGGT
rsmX down F	ACCGGGCTTTGTGTTTAATCTCCGGCATTTCGTGCCTG
rsmX down R	CCCAAGCTTTGCTCAAGGGCTGCGATTA (*Hin*dIII)
rsmX out F	AGATTGCCCTGGATGAAC
rsmX out R	GTGTCCACGTGGAGCACC
rsmY up F	ACGCGTCGACATTACCCAGATCTACGAGGGC (*Sal*I)
rsmY up R	ACAAAAGAAAACCCCGCCGTAGATTAGCTTCAGCCGTC
rsmY down F	GACGGCTGAAGCTAATCTACGGCGGGGTTTTCTTTTGT
rsmY down R	CCCAAGCTTGCATACGCTGTACAACCA (*Hin*dIII)
rsmY out F	CTGGAAACCACGGTTCAT
rsmY out R	AAGAAGGTCATGTGGGAC
rsmZ up F	ACGCGTCGACATCGATCAGTGGCTTTCC (*Sal*I)
rsmZ up R	ATCAAGGATCTCGAACGCGCAAGAGCAATCGCACTA
rsmZ down F	TAGTGCGATTGCTCTTGCGCGTTCGAGATCCTTGAT
Δ*rsmZ* down R	CCCAAGCTTGCCACCCACTATTTCGAA (*Hin*dIII)
rsmZ out F	GCCCGGATTCGGACAAGA
rsmZ out R	GTGCTGATGGACGAAGTG
rsmA up F	ACGCGTCGACGATCGTGCAGAACGTTTC (*Sal*I)
rsmA up R	ATGGCTTGGTTCTTCGTCCACCGACGAGTCAGAATC
rsmA down F	GATTCTGACTCGTCGGTGGACGAAGAACCAAGCCAT
rsmA down R	CCCAAGCTTACCACAACTGGTTGAAGC (*Hin*dIII)

rsmA out F	GAATCCATGGAACAGCCG
rsmA out R	CACAGGCATTGCATTCGA
rsmE up F	ACGCGTCGACTCCACGGTCACCACATTG (*Sal*I)
rsmE up R	CGTGTGTCGGGAATCAACAGCTTTGTAGGGCACCAG
rsmE down F	CTGGTGCCCTACAAAGCTGTTGATTCCCGACACACG
rsmE down R	CCCAAGCTTCCCTGCTCATGGTGTTGA (*Hin*dIII)
rsmE out F	CGATGACCGTGACTTCGA
rsmE out R	TCATCCTGTTGCTGGAGG
luxR1 up F	ACGCGTCGACTATGTCACCCGAAACCCGGA (*Sal*I)
luxR1 up R	CCAGGGGCGTGCGGGCGGTTATCCTTATCATCCTTGATCGA
luxR1 down F	TCGATCAAGGATGATAAGGATAACCGCCCGCACGCCCCTGG
luxR1 down R	CCCAAGCTTGTAGCTGGAGCTGATCAGTG (*Hin*dIII)
luxR1 out F	AACCTTGACGCCGGGAAA
luxR1 out R	GGGTCCTGGAACAGTCCC
luxR2 up F	ACGCGTCGACATCTCGCTGAACATCGACAA (*Sal*I)
luxR2 up R	TTACAGGGCCAAGGCCGGACATTTCATCCATGAATGTGAA
luxR2 down F	TTCACATTCATGGATGAAATGTCCGGCCTTGGCCCTGTAA
luxR2 down R	CCCAAGCTTTGATTTCCCTGGTGGTGGGG (*Hin*dIII)
luxR2 out F	TGATGATGACCTTCGGTGAAA
luxR2 out R	AAGAAGCTGATGGAGCAGC
Construct plasmids	
gacA F (pBBR)	CCCAAGCTTGTTGATAAGGGTGCTAGTAGT (*Hin*dIII)
gacA R (pBBR)	CGCGGATCCTCAGAGGCTGGCATCAACC (*Bam*HI)
pBBR JD F	GTTTTCCCAGTCACGAC
pBBR JD R	CAGGAAACAGCTATGAC
pME6015 JD F	CAAAGCCACGTTGTGTCTCA
pME6015 JD R	GATTCCGACTCGTCCAACAT
prsmX-lux F (pME6522)	CCGGAATTCCACCGACCCTGTGCGCTTTG (*Eco*RI)
prsmX-lux R (pME6522)	AACTGCAGAAAGAAGATTAAACACAAAG (*Pst*I)
prsmY-lux F (pME6522)	CCGGAATTCATTTGGCTATACCGCCTA (*Eco*RI)
prsmY-lux R (pME6522)	AACTGCAGTAGATTAGCTTCAGCCG (*Pst*I)
prsmZ-lux F (pME6522)	CCGGAATTCAAGCGCCTTCGGGAAAT (*Eco*RI)
prsmZ-lux R (pME6522)	AACTGCAGTGATATTAGAGAGTTCCC (*Pst*I)
pluxR1-lux F (pME6015)	CCGGAATTCAAGGGCTTGACCCAGAGCCTT (*Eco*RI)

pluxR1-lux R (pME6015)	AACTGCAGCTGCGGGTGGCCGGAGCTGGAAATCAACT (*Pst*I)
pluxR2-lux F (pME6015)	CCGGAATTCCTTTGGTGTGCCATGGAT (*Eco*RI)
pluxR2-lux R (pME6015)	AACTGCAGGGCGATGACCTTACCAAT (*Pst*I)
pofa-lux F (pME6015)	CCGGAATTCACCGGGGCCGGAACTGTCCGGGTTT (*Eco*RI)
pofa-lux R (pME6015)	AACTGCAGCTGTACCGGACCGGCGTTCATG (*Pst*I)
pluxR1-lacZ F (pME6015)	CCGGAATTCATCCTTCTCATGTGATGGGC (*Eco*RI)
pluxR1-lacZ R (pME6015)	AACTGCAGATACTGCTGGTCAGACTCATA (*Pst*I)
pluxR2-lacZ F (pME6015)	CCGGAATTCTGTCACGAACGCATCATGCA (*Eco*RI)
pluxR2-lacZ R (pME6015)	AACTGCAGCGGCTGCCGGTGCTGGCGATG (*Pst*I)
pofa-lacZ F (pME6015)	CCGGAATTCTGCCTGGCGTCTGTAGGGGGAAA (*Eco*RI)
pofa-lacZ R (pME6015)	AACTGCAGCTGTACCGGACCGGCGTTCATG (*Pst*I)
luxR1-F (pME6032)	CCGGAATTCATGAGTCTGACCAGCAGTAT (*Eco*RI)
luxR1-R (pME6032)	CGGGGTACCTCAGGCGCCGACCATCCAC (*Kpn*I)
luxR2-F (pME6032)	CCGGAATTCATGAACTCACAAATACTGTTTC (*Eco*RI)
luxR2-R (pME6032)	CGGGGTACCTCAGGCCGTCGGGGTTTCG (*Kpn*I)
pME6032 JD F	ACCCTCACTGATCCGCTA
pME6032 JD R	CATCATAACGGTTCTGGCA
pK18mobsacB JD F	TGAGCGCAACGCAATTAAT
pK18mobsacB JD R	GTGAAGCTAGCTTATCGCCA
p-luxR1-F (pBBR)	CCCAAGCTTATTTAAGCATCGGCCCCAG (*Hin*dIII)
p-luxR1-R (pBBR)	ACGCGTCGACTCAGGCGCCGACCATCCAC (*Sal*I)
p-luxR2-F (pBBR)	GTCGACGGTATCGATAAGCTTCTTTGGTGTGCCATGGATGG (*Hin*dIII)
p-luxR2-R (pBBR)	CGCTCTAGAACTAGTGGATCCTCAGGCCGTCGGGGTTTC (*Bam*HI)
luxR1-F (pBBR)	CCCAAGCTTATGAGTCTGACCAGCAGTAT (*Hin*dIII)
luxR1-R (pBBR)	ACGCGTCGACTCAGGCGCCGACCATCCA (*Sal*I)
luxR2-F (pBBR)	CCCAAGCTTATGAACTCACAAATACTGTTTCCA (*Hin*dIII)
luxR2-R (pBBR)	ACGCGTCGACTCAGGCCGTCGGGGTTTCGCT (*Sal*I)
rsmX-F (pBBR)	GTCGACGGTATCGATAAGCTTAAAAAAACCCGCCGAAGCGGG (*Hin*dIII)
rsmX-R (pBBR)	GCTCTAGAACTAGTGGATCCACTAGGGGTTTGCGTAAGTGCG (*Bam*HI)
rsmY-F (pBBR)	GTCGACGGTATCGATAAGCTTAAAACCCCGCCGAAGCGGG (*Hin*dIII)
rsmY-R (pBBR)	GCTCTAGAACTAGTGGATCCCCAGCAAGGGCGTTTGTAAGTC (*Bam*HI)
rsmZ-F (pBBR)	GGTCGACGGTATCGATAAGCTTAAAAAAAGGGGCGGTATGAC (*Hin*dIII)
rsmZ-R (pBBR)	CCGCTCTAGAACTAGTGGATCCGAGAAGTTGGCTTAACTCTCAAC (*Bam*HI)
$rsmY^{\Delta GGA}$ up F (pBBR)	GAAAAGTGCCACCTGGCGGCGTTGTGACAATTTACCGAAC

rsmY$^{\Delta GGA}$ up R (pBBR)	GGACACGTAGGATTCCGCCAGAACAGTCTGCAAAGCCCC
rsmY$^{\Delta GGA}$ down F (pBBR)	CTGGCGGAATCCTACGTGTCC
rsmY$^{\Delta GGA}$ down R (pBBR)	CGCCGCCAGGTGGCACTTTTCG
rsmZ$^{\Delta GGA}$ up F (pBBR)	CATTTCCCCGAAAAGTGCCAC
rsmZ$^{\Delta GGA}$ up R (pBBR)	CGCAGGAAGCGATTCATCAGGAAAAATGTGGGCGGGTCAT
rsmZ$^{\Delta GGA}$ down F (pBBR)	CTGATGAATCGCTTCCTGCG
rsmZ$^{\Delta GGA}$ down R (pBBR)	GTGGCACTTTTCGGGGAAATGTGCGCGCCCGCGTTCCTGC
pluxR1-GFP up F (pME6015-GFP)	ACGGGAAACGTCTTGCTCGAGTTATTTGTATAGTTCATCCATGCCATG (*Xho*I)
pluxR1-GFP up R (pME6015-GFP)	GGATATGAGTAAAGGAGAAGAACTTTTCACTGGA
pluxR1-GFP down F (pME6015-GFP)	CTTCTCCTTTACTCATATCCTTATCATCCTTGATCGAGA
*pluxR1**-GFP down F (pME6015-GFP)	CTTCTCCTTTACTCATATCCTTATCATCTTTGATCGAGA
pluxR1-GFP down R (pME6015-GFP)	CGGGGATCCGTCGACCTGCAGAAGGGCTTGACCCAGAGCC (*Pst*I)
pluxR2-GFP up F (pME6015-GFP)	ACGGGAAACGTCTTGCTCGAGTTATTTGTATAGTTCATCCATGCCATG (*Xho*I)
pluxR2-GFP up R (pME6015-GFP)	GATGAGTAAAGGAGAAGAACTTTTCACTGGA
pluxR2-GFP down F (pME6015-GFP)	GTTCTTCTCCTTTACTCATCATTTCATCCATGAATGTGAA
*pluxR2**-GFP down F (pME6015-GFP)	GTTCTTCTCCTTTACTCATCATTTCATGGATGAATGTGAA
pluxR2-GFP down R (pME6015-GFP)	CGGGGATCCGTCGACCTGCAGCTTTGGTGTGCCATGGATGG (*Pst*I)
pofaA-GFP up F (pME6015-GFP)	ACGGGAAACGTCTTGCTCGAGTTATTTGTATAGTTCATCCATGCCATG (*Xho*I)
pofaA-GFP up R (pME6015-GFP)	GTCCGGTACAGAGTAAAGGAGAAGAACTTTTCACTGGA
pofaA-GFP down F (pME6015-GFP)	TCCTTTACTCTGTACCGGACCGGCGTT
pofaA-GFP down R (pME6015-GFP)	CGGGGATCCGTCGACCTGCAGTTCAGCTGATCAGCAGAGCTGC (*Pst*I)
pluxR1$^{\Delta P1\text{-}2}$-GFP up F (pME6015-GFP)	ACGGGAAACGTCTTGCTCGAGTTATTTGTATAGTTCATCCATGCCATG (*Xho*I)
pluxR1$^{\Delta P1\text{-}2}$-GFP up R (pME6015-GFP)	CCTGGCAGTAAAGGAGAAGAACTTTTCACTGGA
pluxR1$^{\Delta P1\text{-}2}$-GFP down F (pME6015-GFP)	TCTTCTCCTTTACTGCCAGGGCCTCGCCAAGG
pluxR1$^{\Delta P1\text{-}2}$-GFP down R (pME6015-GFP)	CGGGGATCCGTCGACCTGCAGTCAGCTGAATCGGCTGTAGGA (*Pst*I)
pofaA$^{\Delta P4}$-GFP up F (pME6015-GFP)	ACGGGAAACGTCTTGCTCGAGTTATTTGTATAGTTCATCCATGCCATG (*Xho*I)
pofaA$^{\Delta P4}$-GFP up R (pME6015-GFP)	CCGCCAGTAAAGGAGAAGAACTTTTCACTGGA
pofaA$^{\Delta P4}$-GFP down F (pME6015-GFP)	TTCTTCTCCTTTACTGGCGGGCAGTGGCTGGCT
pofaA$^{\Delta P4}$-GFP down R (pME6015-GFP)	CGGGGATCCGTCGACCTGCAGCAGAGCTGCTGCAACAGCC (*Pst*I)
luxR1-GFP up F (pME6015-GFP)	ACGGGAAACGTCTTGCTCGAGTTATTTGTATAGTTCATCCATGCCATG (*Xho*I)
luxR1-GFP up R (pME6015-GFP)	GCGCCAGTAAAGGAGAAGAACTTTTCACTGGA
luxR1-GFP down F (pME6015-GFP)	TTCTTCTCCTTTACTGGCGCCGACCATCCACTT
luxR1-GFP down R (pME6015-GFP)	CGGGGATCCGTCGACCTGCAGAAGGGCTTGACCCAGAGCC (*Pst*I)
luxR2-GFP up F (pME6015-GFP)	ACGGGAAACGTCTTGCTCGAGTTATTTGTATAGTTCATCCATGCCATG (*Xho*I)

luxR2-GFP up R (pME6015-GFP)	ACGGCCAGTAAAGGAGAAGAACTTTTCACTGGA
luxR2-GFP down F (pME6015-GFP)	TCTTCTCCTTTACTGGCCGTCGGGGTTTCGCT
luxR2-GFP down R (pME6015-GFP)	CGGGGATCCGTCGACCTGCAGCTTTGGTGTGCCATGGATGG (*Pst*I)
Protein expression	
rsmA-F (pET28a)	CCGGAATTCATGCTGATTCTGACTCGTCGG (*Eco*RI)
rsmA R (pET28a)	ACGCGTCGACTTAATGGCTTGGTTCTTCGTC (*Sal*I)
rsmE F (pET28a)	CCGGAATTCATGCTGATACTCACCCGC (*Eco*RI)
rsmE R (pET28a)	ACGCGTCGACTCAGGGGGTTTCGCGTTT (*Sal*I)
luxR1-F (pOPTHis)	GGGAATTCCATATGAGTCTGACCAGCAGTATTGCG (*Nde*I)
luxR1-R (pOPTHis)	CGCGGATCCTCAGGCGCCGACCATCCA (*Bam*HI)
luxR2-F (pOPTHis)	GGGAATTCCATATGAACTCACAAATACTGTTTCCA (*Nde*I)
luxR2-R (pOPTHis)	CGCGGATCCTCAGGCCGTCGGGGTTTC (*Bam*HI)
pOPTHis JD F	GATGCGCCGGGTTATGAA
pOPTHis JD R	ACTCAGCTTCCTTTCGGG
RNA-EMSA analysis	
rsmX F (T7)	TAATACGACTCACTATAGGGTTTCCCACAACTGCAGCGC
rsmX R (T7)	AAAAAAACCCGCCGAAGCGGGTT
rsmY F (T7)	TAATACGACTCACTATAGGGATGGACGTCGCGCAGGAAGC
rsmY R (T7)	AAAACCCCGCCGAAGCGGG
rsmZ F (T7)	TAATACGACTCACTATAGGGTGTCGACGGATAGACACAGCC
rsmZ R (T7)	AAAAAAAGGGGCGGTATGACC
luxR1 5′UTR	CUCGAUCAAGGAUGAUAAGG
luxR2 5′UTR	ACAUUCAUGGAUGAAAUG
ofaABC 5′UTR	CCAGCCAGCCACUGCCCGCC
DNA-EMSA analysis	
luxR1-F (Biotin)	TTCCACCGCAGGCTTGCC
luxR1-R (Biotin)	GTTGCAGCATTTCGCTGGC
luxR1-F	TTCCACCGCAGGCTTGCC
luxR1-R	GTTGCAGCATTTCGCTGGC
ofaA-F (Biotin)	CGTTCACGCGTAGCCAATT
ofaA-R (Biotin)	TACCGGACCGGCGTTCATG
ofaA-F	CGTTCACGCGTAGCCAATT
ofaA-R	TACCGGACCGGCGTTCATG
luxR1$^{\Delta P1\text{-}2}$-F (FAM）	GCCAGGGCCTCGCCAAGGCT

luxR1$^{\Delta P1-2}$-R	TCAGCTGAATCGGCTGTAGG
ofaA$^{\Delta P4}$-F (FAM）	GGCGGGCAGTGGCTGGCTGG
ofaA$^{\Delta P4}$-R	CAGAGCTGCTGCAACAGCCC
MST analysis	
luxR1-F (FAM)	TTCCACCGCAGGCTTGCC
luxR1-R (FAM)	GTTGCAGCATTTCGCTGGC
ofaA-F (FAM)	CGTTCACGCGTAGCCAATT
ofaA-R (FAM)	TACCGGACCGGCGTTCATG
ChIP-qPCR analysis	
luxR1-P1-F	TCGAGACAAATCGCTGATCG
luxR1-P1-R	TTAAATCAACGCCCGGGAAC
luxR1-P2-F	AGTTCCCGGGCGTTGATTTAAG
luxR1-P2-R	GCCATGATGTCCCGGTTTCC
luxR1-P3-F	CCAAGGCTCTGGGTCAAGC
luxR1-P3-R	AACCTTGACGCCGGGAAAC
luxR1-P4-F	TTTCCCGGCGTCAAGGTTG
luxR1-P4-R	GAATCGGCTGTAGGAAATTGGC
ofaA-P1-F	GGCCCATCACATGAGAAGGATC
ofaA-P1-R	TGACTGGCGCGACGAAAG
ofaA-P2-F	TGGGGCCGATGCTTAAATCC
ofaA-P2-R	CGGCGGAAAAATGCGTCATAAG
ofaA-P3-F	ATTTCCTACAGCCGATTCAGC
ofaA-P3-R	GCCTGTATGTCACCCGAAAC
ofaA-P4-F	TTTCCCGGCGTCAAGGTTG
ofaA-P4-R	GAATCGGCTGTAGGAAATTGGC
qRT-PCR analysis	
RT *luxR1* F	AAACCCCGCACTTCTACTTC
RT *luxR1* R	GTTCGTCCAGGGTCCATTC
RT *luxR2* F	ACATTGGTAAGGTCATCGCC
RT *luxR2* R	CAGTTGGGTGATATGGGTGG
RT *ofaA* F	TCAACTATCGCCACAACCTC
RT *ofaA* R	GTAGCTGAGCACCTCTTCG
RT *gyrB* F	AGAGCGGATGAAGATCGTTG
RT *gyrB* R	GGAAGGACAGTTCACGGATC
RT *rsmX* F	TCAGGATCAGGGAAGGTCG

RT *rsmX* R	CGAAGCGGGTTTTTTCCAAG
RT *rsmY* F	CAAAGCAACAACACGGACAC
RT *rsmY* R	CAGACTGTTTCCCTGACATCC
RT *rsmZ* F	CGGATAGACACAGCCATCAAG
RT *rsmZ* R	TCCCTGTGTTCCTTTTCATCG

Restriction sites used for cloning are underlined in primers.

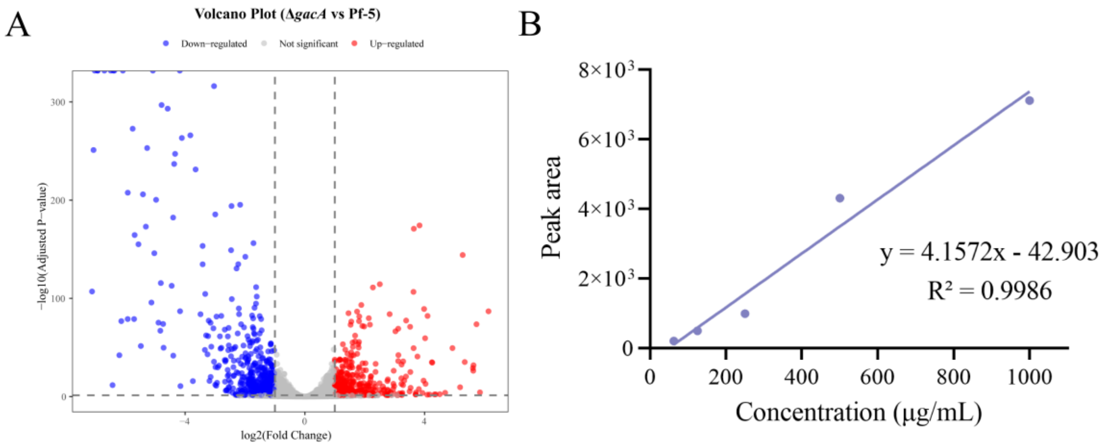

Supplementary Figure 1. (A) Volcano plot of DEGs between Pf-5 and Δ*gacA*. Each point represents a single gene (|log2(Fold Change)| >1, FDR < 0.05). A total of 982 DEGs were identified, including 467 upregulated (red) and 515 downregulated genes (blue). Three replicates were done for each experiment. Source data are provided as a Source Data. (B) The standard curve of orfamide A standards by HPLC analysis.

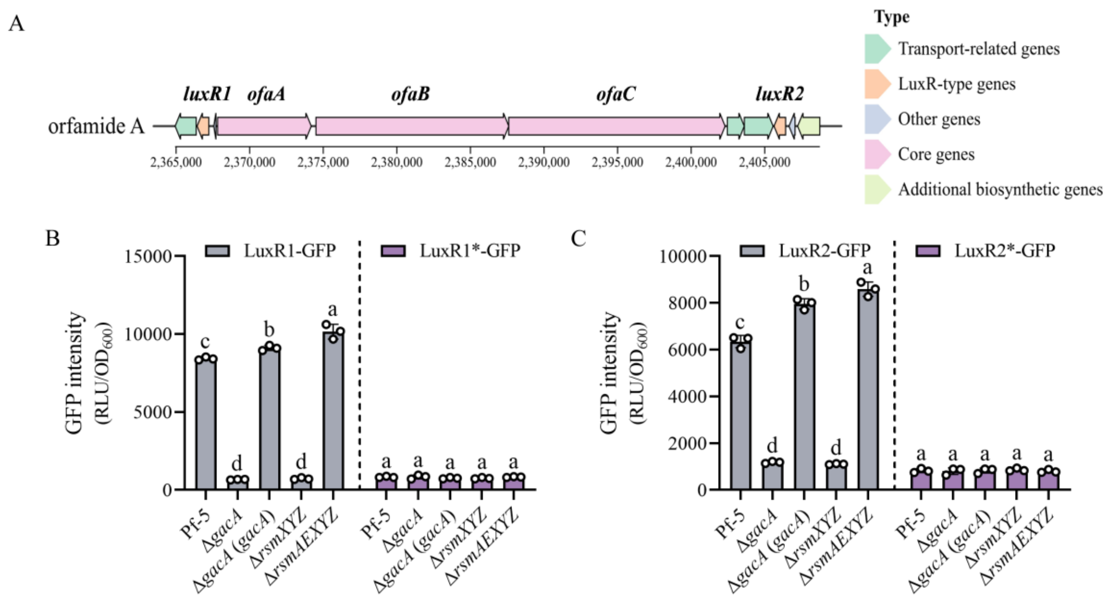

Supplementary Figure 2. (A) Schematic of the gene cluster encoding Orfamide A and LuxR-

type transcriptional regulators in Pf-5. Names were provided on the top of each gene. The *ofaABC* gene cluster (*PFL_RS10840-10850*) was indicated in pink, and *luxR1* and *luxR2* genes (*PFL_RS10835* and *PFL_RS10865*, respectively) were indicated in light green. (B-C) GFP intensity of *luxR1* and *luxR2* promoters in different strains at 24 h using the translational P_{luxR1}::*gfp* (B) and P_{luxR2}::*gfp* (C) fusions. LuxR1*, the GGA motif of *luxR1* has been converted to AGA. LuxR2*, the GGA motif of *luxR2* has been converted to CCA. Data were presented as the mean ± SD from three independent experiments (n=3) with similar results. Different lowercase letters within the same row indicate statistically significant differences.

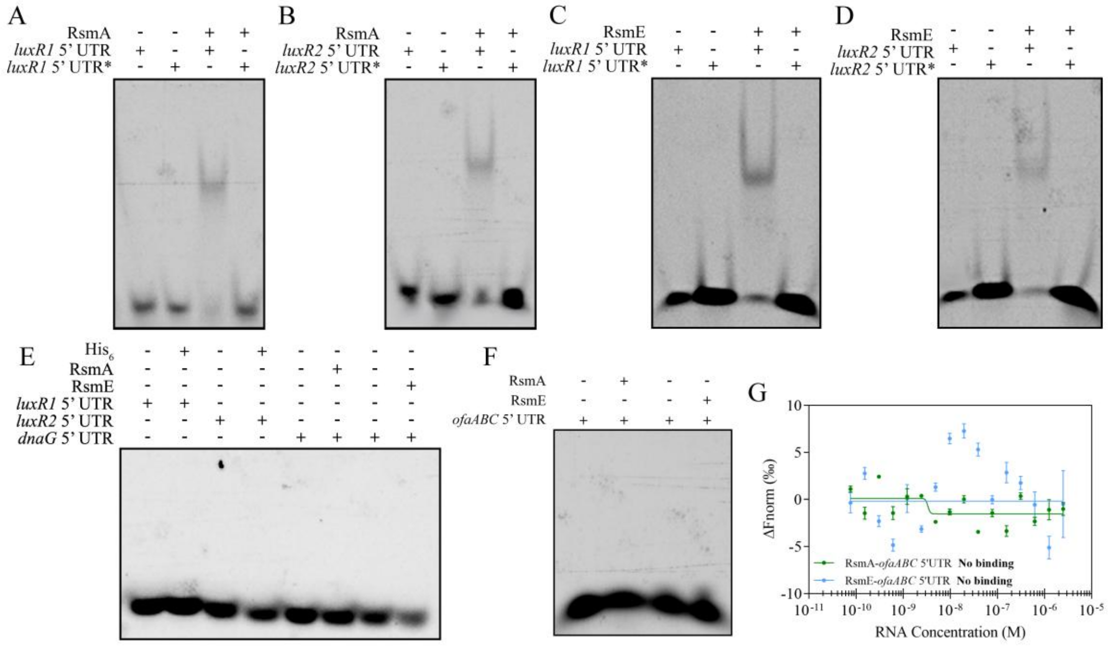

Supplementary Figure 3. (A-D) The RNA-EMSA experiments showed that RsmA (A-B) and RsmE (C, D) protein interacted with *luxR1* and *luxR2* 5′UTR, and substitution of the Rsm binding motif of *luxR1* 5′UTR (GGA to AGA) and *luxR2* 5′UTR (GGA to CCA) abolished the binding of RsmA (A-B) and RsmE (C, D) with *luxR1* and *luxR2* 5′UTR. (E) The RNA-EMSA experiments showed that there was no interaction between His_6 protein with *luxR1* and *luxR2* 5′UTR, and RsmA/E with *dnaG* 5′UTR. (F-G) The RNA-EMSA and MST experiments showed that there was no binding between RsmA and RsmE with *ofaABC* 5′UTR. All blots shown were representative of three separate experiments with similar results. MST assays were presented as the mean ± SD from three independent experiments (n=3) with similar

results.

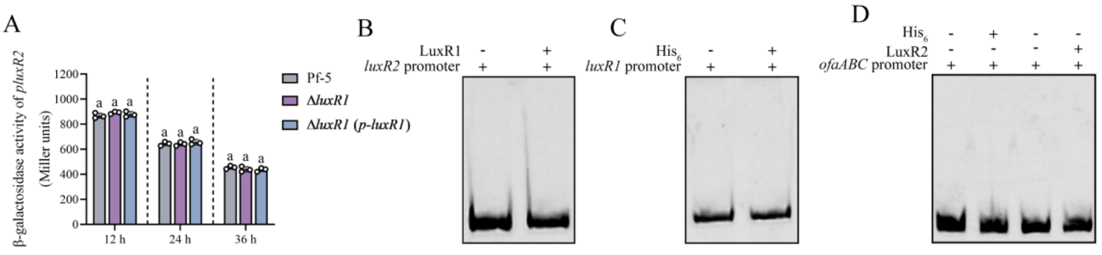

Supplementary Figure 4. (A) β-galactosidase activity of *luxR2* promoter activity using the transcriptional P_{luxR2}::*lacZ* fusion reporter expressed in Pf-5, Δ*luxR1* and Δ*luxR1* (*p-luxR1*) at 12 h, 24 h and 36 h. Data were presented as the mean ± SD from three independent experiments (n=3) with similar results. Different lowercase letters within the same row indicate statistically significant differences. (B) EMSA experiment showed that there was no interaction between LuxR1 protein with *luxR2* promoter. (C) EMSA experiment showed that there was no interaction between His_6 protein with *luxR1* promoter. (D) EMSA experiment showed that there was no interaction between His_6 protein with *ofaABC* promoter, and LuxR2 protein with *ofaABC* promoter. All blots shown in B-F were representative of three separate experiments with similar results.

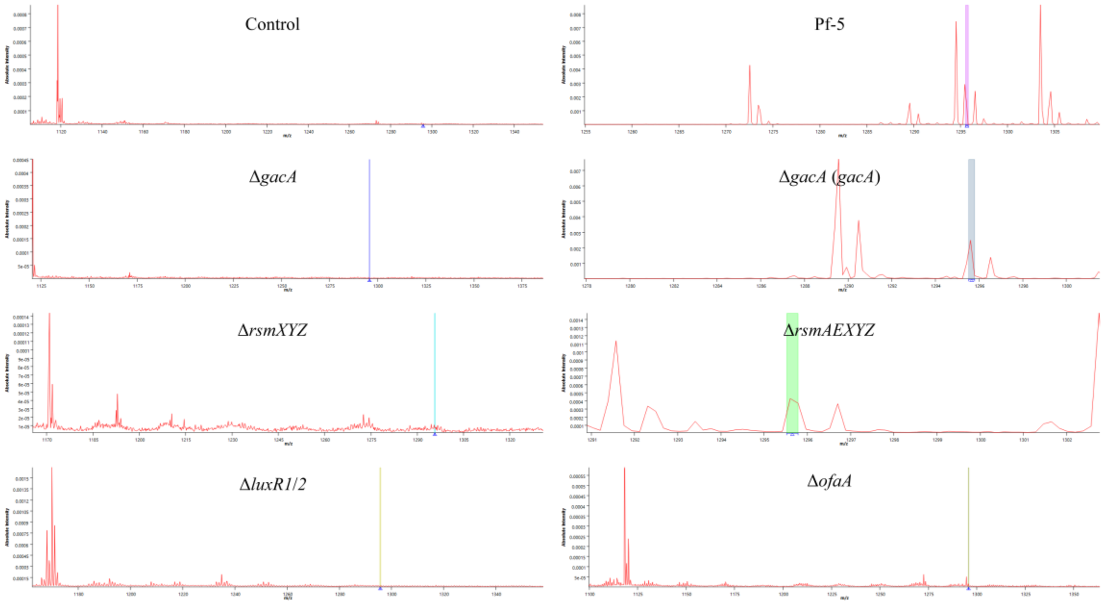

Supplementary Figure 5. MALDI-MSI mass spectra of orfamide A in the tomato roots. Pf-5, Δ*gacA* (*gacA*) and Δ*rsmAEXYZ* produced orfamide A in the tomato rhizosphere, while Δ*gacA*, Δ*rsmXYZ*, Δ*luxR1/2* and Δ*ofaA* did not. The MSI results were repeated for at least

three times.

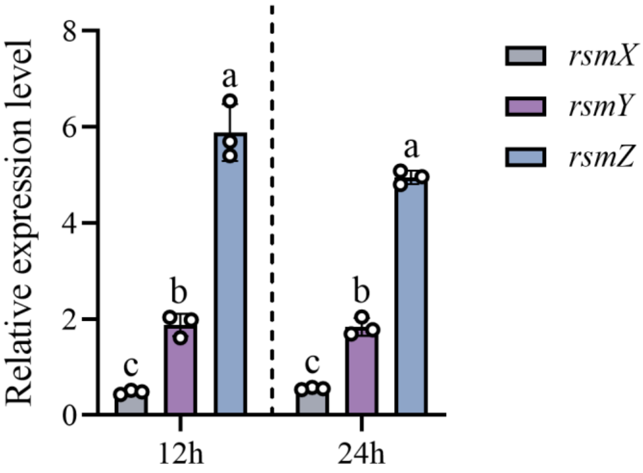

Supplementary Figure 6. qRT-PCR analysis of *rsmX*, *rsmY* and *rsmZ* mRNA levels in Pf-5. The comparative cycle threshold (CT) method was used for data analysis and relative fold difference was estimated as $2^{-\Delta CT}$. Data were presented as the mean ± SD from three independent experiments (n=3) with similar results. Different lowercase letters within the same row indicate statistically significant differences.
